# Loss of cholinergic receptor muscarinic 1 impairs cortical mitochondrial structure and function: implications in Alzheimer’s disease

**DOI:** 10.3389/fcell.2023.1158604

**Published:** 2023-05-18

**Authors:** Mohammad Golam Sabbir, Mamiko Swanson, Benedict C. Albensi

**Affiliations:** ^1^ Division of Neurodegenerative Disorders, St. Boniface Hospital Albrechtsen Research Centre, Winnipeg, MB, Canada; ^2^ Alzo Biosciences Inc, SanDiego, CA, United States; ^3^ Canadian Centre for Agri-Food Research in Health and Medicine, St. Boniface Hospital Albrechtsen Research Centre, Winnipeg, MB, Canada; ^4^ Barry & Judy Silverman College of Pharmacy, Nova Southeastern University, Fort Lauderdale, FL, United States; ^5^ Department of Pharmacology & Therapeutics, University of Manitoba, Winnipeg, MB, Canada

**Keywords:** cholinergic receptor muscarinic 1, mitochondria, pyramidal neuron, oligomerization, Alzheimer’s disease, respiration, ATP synthase, transmission electron microscopy

## Abstract

**Introduction:** Cholinergic Receptor Muscarinic 1 (CHRM1) is a G protein-coupled acetylcholine (ACh) receptor predominantly expressed in the cerebral cortex. In a retrospective *postmortem* brain tissues-based study, we demonstrated that severely (≥50% decrease) reduced CHRM1 proteins in the temporal cortex of Alzheimer’s patients significantly correlated with poor patient outcomes. The G protein-mediated CHRM1 signal transduction cannot sufficiently explain the mechanistic link between cortical CHRM1 loss and the appearance of hallmark Alzheimer’s pathophysiologies, particularly mitochondrial structural and functional abnormalities. Therefore, the objective of this study was to analyze the molecular, ultrastructural, and functional properties of cortical mitochondria using CHRM1 knockout (Chrm1^-/-^) and wild-type mice to identify mitochondrial abnormalities.

**Methods:** Isolated and enriched cortical mitochondrial fractions derived from wild-type and Chrm1^-/-^ mice were assessed for respiratory deficits (oxygen consumption) following the addition of different substrates. The supramolecular assembly of mitochondrial oxidative phosphorylation (OXPHOS)-associated protein complexes (complex I-V) and cortical mitochondrial ultrastructure were investigated by blue native polyacrylamide gel electrophoresis and transmission electron microscopy (TEM), respectively. A cocktail of antibodies, specific to Ndufb8, Sdhb, Uqcrc2, Mtco1, and Atp5a proteins representing different subunits of complexes I-V, respectively was used to characterize different OXPHOS-associated protein complexes.

**Results:** Loss of Chrm1 led to a significant reduction in cortical mitochondrial respiration (oxygen consumption) concomitantly associated with reduced oligomerization of ATP synthase (complex V) and supramolecular assembly of complexes I-IV (Respirasome). Overexpression of Chrm1 in transformed cells (lacking native Chrm1) significantly increased complex V oligomerization and respirasome assembly leading to enhanced respiration. TEM analysis revealed that Chrm1 loss led to mitochondrial ultrastructural defects and alteration in the tinctorial properties of cortical neurons causing a significant increase in the abundance of dark cortical neurons (Chrm1^-/-^ 85% versus wild-type 2%).

**Discussion:** Our findings indicate a hitherto unknown effect of Chrm1 deletion in cortical neurons affecting mitochondrial function by altering multiple interdependent factors including ATP synthase oligomerization, respirasome assembly, and mitochondrial ultrastructure. The appearance of dark neurons in Chrm1^-/-^ cortices implies potentially enhanced glutamatergic signaling in pyramidal neurons under Chrm1 loss condition. The findings provide novel mechanistic insights into Chrm1 loss with the appearance of mitochondrial pathophysiological deficits in Alzheimer’s disease.

## 1 Introduction

Muscarinic acetylcholine (ACh) receptors (mAChR) constitute a subfamily of metabotropic G protein-coupled receptors (GPCR) activated by the endogenous neurotransmitter ACh. Five subtypes of mAChRs, M1-M5 (encoded by the CHRM1-5 genes, respectively), are widely expressed in a spatiotemporal manner in non-neuronal peripheral tissues (Wessler and Kirkpatrick, 2008) as well as in the neurons and glial cells of the central and peripheral nervous systems (CNS and PNS) (Gong et al., 2003; Eglen, 2005; Wess et al., 2007). The mAChRs in the CNS regulate cognition, behavior, sensory, motor, and autonomic processes (Coyle et al., 1983; Bartus, 2000; Messer, 2002; Fisher et al., 2003; Eglen, 2005; Wess et al., 2007; Matera and Tata, 2014). Altered mAChR expression and function has been described in several neurodegenerative diseases such as Alzheimer’s disease (AD) (Tata et al., 2014a; Tata et al., 2014b; Petrova et al., 2020). The degeneration of cholinergic neurons and cholinergic hypofunction are identified as pathologies associated with AD (Nilsson et al., 1986; Pavia et al., 1998; Jiang et al., 2014) leading to the establishment of the “cholinergic hypothesis” of Alzheimer’s pathogenesis (Bartus et al., 1982). Among the different mAChR subtypes, CHRM1 is more abundantly expressed in the cerebral cortex (Giraldo et al., 1987; Ehlert and Tran, 1990; Levey, 1996). On the other hand, neuropathological studies demonstrated that pathological hallmarks of AD begin to manifest in the cortical region of the brain during the progression of the disease (Braak and Braak, 1991; Palmqvist et al., 2017). These observations led us to perform a recent study analyzing the abundance of CHRM1 protein in a large cohort of *postmortem* human brain tissues, specifically temporal cortices, derived from AD (*N* = 74) and clinicopathologically normal individuals (N = 19) (Sabbir et al., 2022). Our study revealed loss of CHRM1 protein in the temporal cortices of a subset of AD patients that was significantly associated with poor survival, i.e., early death occurred <65-75 years (Sabbir et al., 2022). This interesting finding renewed our interest in understanding the molecular and physiological effect of CHRM1 loss in the cerebral cortex and its link to AD pathogenesis.

The mAChR signal transduction pathway involves several well-characterized components including enzymes and ion channels (Lanzafame et al., 2003). CHRM1 signals through G_q/11_, activates phospholipase C, which initiates the phosphatidylinositol turnover response (Haga, 2013). This leads to the inositol trisphosphate-mediated release of calcium ions (Ca^2+^) from the endoplasmic reticulum and diacylglycerol-mediated activation of protein kinase C (Haga, 2013). CHRM1 also signals through Gs alpha, producing the second messenger cyclic adenosine monophosphate (cAMP) through stimulation of adenylyl cyclase activity, independent of phospholipase C activation (Burford and Nahorski, 1996). As a consequence of these signal transduction events, depending on cell type, other cellular effectors may become activated, for example, mitogen-activated protein kinase (MAPK) (Hamilton and Nathanson, 2001; Sabbir and Fernyhough, 2018). The CHRM1-β-Arrestin-MAPK signaling cascade has the potential to enhance neuronal survival and function by upregulating certain protection systems and/or blockade of apoptosis, or modulation of learning and memory (Fukunaga and Miyamoto, 1998; Peng et al., 2010; Albert-Gascó et al., 2020). Thus, it is conceivable that loss of CHRM1 protein may lead to dysfunctional MAPK signaling underlying the pathogenesis of AD (Zhao et al., 2002). However, despite these advances in our understanding of muscarinic signal transduction, a definitive molecular mechanism linking CHRM1 loss and the appearance of hallmark Alzheimer’s pathology during the progression of the disease is still lacking. One particular pathophysiology is immensely important, mitochondrial abnormalities and malfunction in AD (Wang et al., 2020).

Cortical neurons consume 4.7 billion ATP molecules per second (Zhu et al., 2012) largely via oxidative phosphorylation (OXPHOS) (Karagiannis et al., 2021). It has been estimated that over 80% of the energy in the myelinated neuron is expended by postsynaptic potentials (Alle et al., 2009; Harris et al., 2012). Presynaptic vesicle recycling also acts as an additional energy-consuming process. Therefore, a healthy number of mitochondria is required at the synapse to support neuronal activity not only by providing enough energy supply, but also by minimizing oxidative damage due to the production of mitochondrial reactive oxygen species (ROS). Most cortical neurons express CHRM1 (37). Activation of mAChRs in cortical neurons enhances excitatory synaptic response through the generation of dendritic Ca^2+^ spikes (Häusser et al., 2000), *N*-methyl-D-aspartate (NMDA) spikes (Schiller and Schiller, 2001; Albensi, 2007), and action potential bursts which provide the main source of Ca^2+^ influx necessary to induce synaptic plasticity (Fernández de Sevilla et al., 2021). Mitochondrial function and dynamics in the synapse are tightly regulated by synaptic calcium (Datta and Jaiswal, 2021), therefore it is highly probable that loss of CHRM1 and synaptic plasticity during Alzheimer’s pathogenesis is linked to mitochondrial abnormalities. Disruption of several aspects of mitochondrial functioning (Wang et al., 2020), for example, mitophagy (Mary et al., 2022), bioenergetics (Reiss et al., 2022), proteostasis, and biogenesis as well as morphometric abnormalities (Hirai et al., 2001a) has been reported in AD, but a direct link with cholinergic loss in the cortical neurons has not yet been established. Therefore, the objective of this study is to characterize the molecular, structural, and functional deficits in mitochondria derived from wild-type and Chrm1 deleted (Chrm1^-/-^) transgenic mouse cerebral cortex.

The OXPHOS system is involved in the sequential transfer of electrons from reduced cofactors to molecular oxygen through five multiprotein complexes (MPCs), namely, NADH:ubiquinone oxidoreductase (complex I), succinate-coenzyme Q reductase (complex II), coenzyme Q: cytochrome c-oxidoreductase (complex III), cytochrome c oxidase (complex IV)m and ATP synthase (complex V), facilitating the formation of a proton gradient coupled to the synthesis of ATP in the mitochondria. Each complex is made up of multiple protein subunits (Zhu et al., 2016). Alterations in the relative abundance of complexes I-V-associated subunits has been reported in Alzheimer’s brain tissues, specifically, NADH-Ubiquinone Oxidoreductase Subunit B8 (NDUFB8) (Francis et al., 2014), Succinate Dehydrogenase Complex Iron-Sulfur Subunit B (SDHB) (Alldred et al., 2021), Ubiquinol-Cytochrome C Reductase Core Protein (UQCRC2) (Adav et al., 2019), Mitochondrially Encoded Cytochrome C Oxidase I (MT-CO1) (Hoozemans et al., 2008), and ATP Synthase F1 Subunit Alpha (ATP5F1A) (Misrani et al., 2021), respectively. Therefore, using a cocktail of antibodies specific to these protein subunits, we studied their relative abundance in isolated and enriched cortical mitochondrial fractions derived from wild-type and Chrm1^-/-^ mice. The current concept of mitochondrial architecture states that the OXPHOS-associated protein complexes are not randomly distributed within the inner mitochondrial membrane, but assemble into supramolecular structures (Wittig and Schagger, 2009). For example, the majority of complex I is found bound with a complex III dimer and complex IV (I_1_III_2_IV_1_) (Schäfer et al., 2006; Guo et al., 2017a), a Supercomplex (SC) structure that contains all complexes required to pass electrons from NADH to O_2,_ hence known as a “respirasome” (Vonck and Schäfer, 2009). Some SCs appear to associate with larger structures, or megacomplex (MC), such as a string of dimeric ATP synthase (V_2_) (Bultema et al., 2009). A row-like organization of different stoichiometries of complex I, complex II, complex III, and complex IV is a major physiological module of the respiratory chain (Schäfer et al., 2006) and is key to rapid and efficient electron transfer during oxidative phosphorylation (Lapuente-Brun et al., 2013). Therefore, we studied the supramolecular assembly of complexes I-V in wild-type and Chrm1^-/-^ mice cortical mitochondria using two dimensional blue-native polyacrylamide gel electrophoresis followed by sodium dodecyl-sulfate polyacrylamide gel electrophoresis (2D BN-PAGE/BN-PAGE) (Vonck and Schäfer, 2009). Furthermore, using transmission electron microscopy (TEM), we studied mitochondrial ultrastructure in wild-type and Chrm1^-/-^ mice cortical neurons and neuropil. Overall findings indicate that Chrm1 loss significantly decreased cortical mitochondrial function due to altered mitochondrial abundance, ultrastructure, ATP synthase oligomerization, and respirasome assembly.

## 2 Materials and methods

### 2.1 Chrm1 knockout mouse

The Chrm1 knockout mouse (Chrm1^-/-^) line (Sabbir et al., 2022) was provided by Dr. Jurgen Wess, NIH. All animal procedures followed the guidelines of the University of Manitoba Animal Care Committee using the Canadian Council of Animal Care rules. Adult male mice, 6 months old, were used for isolation of the cerebral cortex tissue.

### 2.2 Cell culture, cloning, and transfection

The mouse tumor cell line, OC-033, was developed by Sabbir et al. as previously described (Sabbir et al., 2016). This cell line was used because it is easy to achieve high (≥80%) transfection efficiency using cationic lipids, such as lipofectamine reagent (catalog number: L3000001, ThermoFisher) and it does not express endogenous Chrm1 (Sabbir and Fernyhough, 2018). The cells were cultivated in Dulbecco’s modified Eagle’s medium (DMEM) supplemented with 10% heat-inactivated FBS and 1X antibiotic antimycotic solution (A5955, Sigma). The full-length rat Chrm1 was cloned in the pEGFP-C1 vector (Clontech, now Takara Bio United States, Inc., Mountain View, CA) as described previously (Sabbir et al., 2018).

### 2.3 Isolation and enrichment of the mitochondria

Enriched mitochondria were from cultured cells isolated by a method previously described by Sabbir et al. (2020); Sabbir et al., 2021). The freshly dissected adult male mouse cerebral cortex tissues were chopped on ice and immersed in an ice-chilled mitochondrial isolation buffer containing 70 mM sucrose, 210 mM mannitol, 5 mM HEPES pH 7.2, and 1 mM EGTA. (Sabbir et al., 2020). The cells/tissues were disrupted by a Teflon Dounce homogenizer and the homogenate was centrifuged at 800 g for 10 min at 4°C. Following centrifugation, the supernatant was decanted through 2 layers of cheesecloth to a separate tube and centrifuged at 8000 g for 10 min at 4°C. After the removal of the supernatant, the pellet was resuspended in the mitochondrial isolation buffer and washed thoroughly and the centrifugation was repeated. The final pellet was resuspended in a minimal volume and its total protein concentration was determined after which it was used for functional assays.

### 2.4 Mitochondrial function test

The coupling and electron flow assays were performed using microgram (5 µg) quantities of isolated enriched mouse cortical mitochondria in a Seahorse XF24 analyzer (Agilent) as described by Sabbir (Sabbir et al., 2021). For the coupling assay, 10 mM succinate and 2 µM rotenone were used as a substrate. ADP (4 mM), oligomycin (2.5 μg/mL), FCCP (4 µM), and antimycin A (4 µM) were then sequentially injected, and measurements of oxygen consumption rate (OCR) were taken after each injection. For the electron flow assay, 10 mM pyruvate, 2 mM malate, and 4 µM FCCP were used as a substrate. In the electron flow assay, 2 µM rotenone, 10 mM succinate, 4 µM antimycin A, 10 mM ascorbate +100 µM TMPD (N, N, N9, N9-Tetramethylp-phenylenediamine) were sequentially injected, and measurements of OCR were taken after each injection. All inhibitor concentrations are described as final concentrations. Mitochondrial function in green fluorescent protein (GFP) and Chrm1-GFP overexpressed cells were measured as described previously (Sabbir, 2019).

### 2.5 Blue-native polyacrylamide gel electrophoresis (BN-PAGE)

The 2D BN-PAGE/SDS-PAGE analysis was performed as described by Sabbir (2018); Sabbir (2019); Sabbir et al. (2021). Briefly, the enriched cortical mitochondrial fraction or cell pellet was lysed in 1X phosphate-buffered Saline (PBS) supplemented with 1X Halt protease and phosphatase inhibitor cocktail (catalog number: 1861281, Thermo Scientific) and 1.5% *n*-Dodecyl β-D-maltoside (catalog number: D4641, Sigma) and sonicated. The proteins were then separated in 4%-15% gradient BN-PAGE. The gel strips (individual lanes) were carefully excised including the 3.2% stacking gel and immersed in the Laemmli sample buffer containing freshly prepared 100 mg/mL dithiothreitol (DTT). The gel slices were incubated in a sample buffer for 30 min at room temperature (RT) and then the proteins in the gel slices were separated in 2^nd^ dimension SDS-PAGE and immunoblotted.

### 2.6 Transmission electron microscopy (TEM)

Adult mice were saline-formaldehyde perfused for 15 min and freshly dissected to harvest tissues. After perfusion, cortical tissues were fixed in glutaraldehyde for 2 h. Alternatively, freshly dissected brain tissues were immediately fixed in 2% glutaraldehyde in Sorenson’s buffer (pH 7.4) for 2 h. All tissue samples were postfixed in osmium tetroxide for an hour and washed thrice with distilled water for 5 min each. The tissue samples were then dehydrated by passing through alcohol gradients and transitional dehydration was performed in propylene oxide for 2 × 15 min. The samples were then infiltrated by plastic: propylene oxide for an hour and finally by full plastic overnight. The embedding in full plastic was performed in flat molds and placed in a jar with desiccant. The polymerization was performed overnight at 70°C. Semithin sections (500 nm) were cut to examine the orientation of the tissues. Finally, ultrathin sections (<90 nm) were cut using a Leica ultramicrotome (Leica EM UCF7) with sections mounted on a copper grid (200 square mesh, Cat: V2200, Canemco Inc.), stained with uranyl acetate and lead citrate, then examined using a JEOL transmission electron microscope (model: JEOL JEM-1010, JEOL United States of America Inc.). The sections were imaged using AMT image capture engine software version V602.580.

Enriched mitochondrial fractions from the cortex were prepared by centrifuging 10 μg of enriched mitochondrial fraction suspended in mitochondrial assay buffer in 96 well microplates for 30 min at 2500 g in a horizontal plate rotor. A circular 0.2 μM nitrocellulose membrane was placed at the bottom of individual wells before placing the enriched mitochondrial fraction. Post-centrifugation, the wells were immersed in glutaraldehyde. After 1 h of incubation, the nitrocellulose membranes containing fixed organelle fraction were placed in fresh 2% glutaraldehyde solution for an additional hour and then processed as described above.

### 2.7 Western blotting (WB) and immune-detection

Relative quantification based on WB of proteins was previously described in detail by Sabbir et al. (2020). The relative abundance of complexes I-V was determined by immunoblotting using a cocktail of 5 antibodies (catalog number: ab110413, Abcam) specific to different subunits of OXPHOS complexes, namely, Ndufb8 (complex I), Sdhb (complex II), Uqcrc2 (complex III), Mtco2 (complex IV), and Atp5a (complex V) respectively.

### 2.8 Statistical analysis

Statistical analysis was performed using Prism version 7.00 (GraphPad Software) as described previously (Sabbir et al., 2018; Sabbir et al., 2020; Sabbir et al., 2021; Sabbir et al., 2022). Differences were considered significant with *p* < 0.05 and throughout the text, if a *p*-value is ≤0.05, ≤0.01, ≤0.001, or ≤0.0001, it was flagged by representation with one, two, three, or four asterisks, respectively.

## 3 Results

### 3.1 Chrm1 gene is expressed in mouse cortical neurons

**FIGURE 1:**
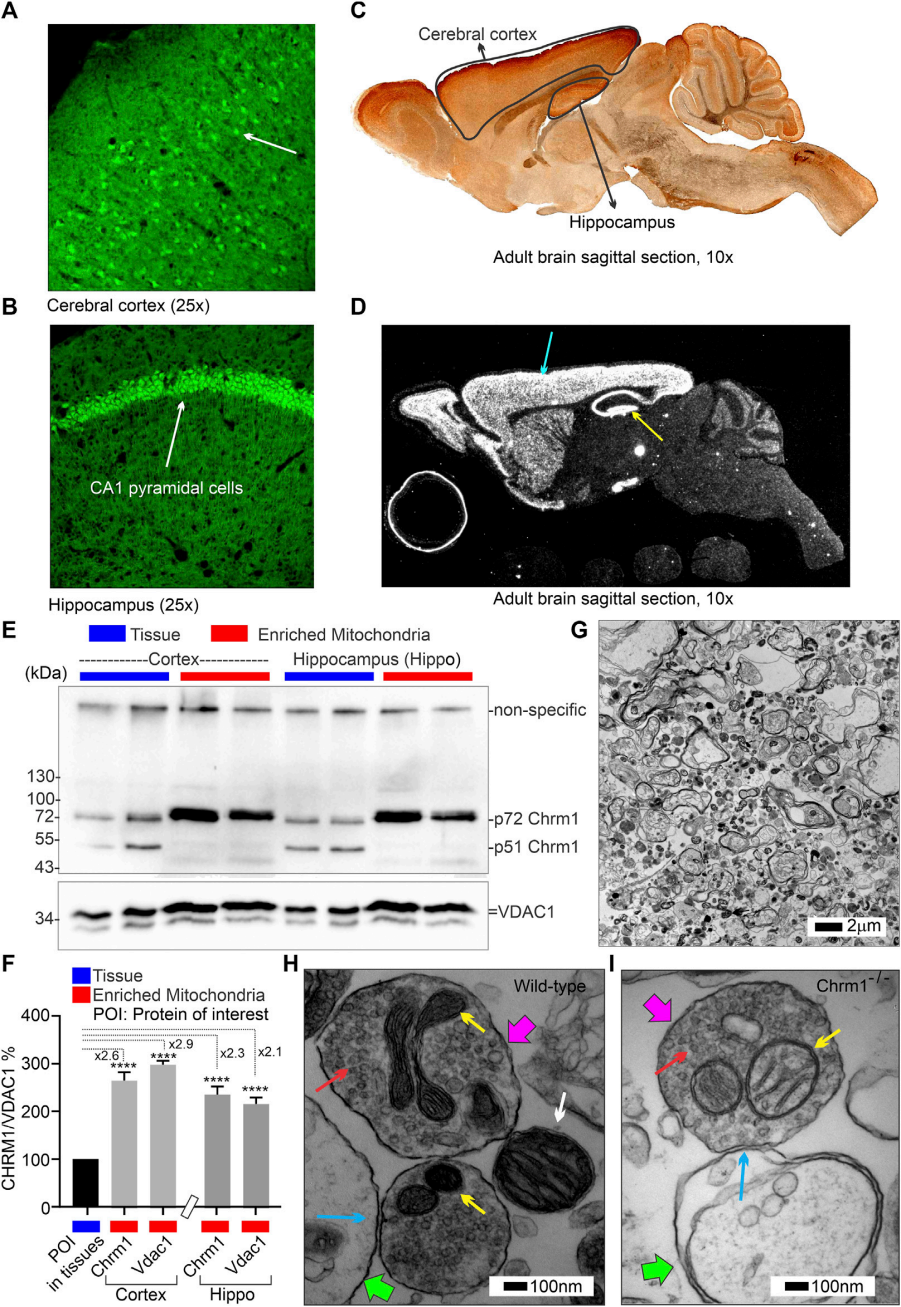
Characterization of Chrm1 protein and synaptic mitochondrial abundance in mouse cortical neurons and enriched cortical mitochondrial fractions. **(A, B)** GENSAT BAC transgenic Chrm1 mouse cerebral cortex and hippocampus sections showing eGFP fluorescence in the pyramidal neurons (white arrows). The eGFP is expressed under the influence of the native Chrm1 promoter. **(C)** Immunohistochemistry image showing eGFP reporter gene expression in adult BAC chrm1 transgenic mouse brain. The intense anti-GFP immunostaining in the black outlined areas indicates Chrm1 abundance in the cortex and hippocampus regions, respectively. **(D)** Image showing the expression of Chrm1 mRNA in the cortex and hippocampus regions of adult mouse brain detected by *in situ* hybridization using a radioactive anti-Chrm1 probe. Data from the BGEM database (Magdaleno et al., 2006). The magnifications of the images presented in **(A–D)** are based on the magnification power of the optical microscope lens used in imaging. **(E)** Immunoblots showing the relative abundance of Chrm1 and Vdac1 in whole tissue lysates and enriched mitochondrial fractions derived from wild-type cortex and hippocampus. **(F)** Bar graph showing the relative amount (%; mean ± SEM) of Chrm1 and Vdac1 proteins in the enriched cortical and hippocampal mitochondrial fractions normalized to the respective protein’s levels in the whole tissue lysates (100%). Two replicates from two independent experiments were used to generate table. *p* values by one-way ANOVA test. **(G)** TEM images showing the ultrastructure of isolated enriched mitochondrial fractions derived from wild-type cortex. **(H and I)** TEM images showing a magnified view of the pre- (pink arrows pointing to the presynaptic membrane) and post-synaptic (green arrows pointing to the postsynaptic membrane) terminals in the enriched fractions containing mitochondria. The yellow arrows are pointing to the synaptic mitochondria, whereas the white arrows are pointing to the isolated unencapsulated mitochondria. Red arrows point to the synaptic vesicles and blue arrows to the synapses (contact between pre and post-synaptic buds). Note the mitochondria in the Chrm1^-/-^ synapse (yellow arrows) has abnormal cristae compared to wild-type.

The Gene Expression Nervous System Atlas (GENSAT) project (Heintz, 2004) created Chrm1 transgenic reporter mouse strain (Founder Line: IO35) by replacing endogenous protein coding sequence with enhanced green fluorescent protein (eGFP) reporter gene using bacterial artificial chromosome (BAC)-based transgenic vectors (Schmidt et al., 2013). The project database (genesat.org) contains histological data from given BAC transgenic mouse lines at different developmental stages including the adult stage; in all cases, the data represent the results of multiple transgenic lines. Sagittal cryosections of the adult mouse brain obtained from the GENSAT database revealed intense GFP fluorescence in the pyramidal neurons (white arrows) of the cortex (Figure 1A) and hippocampus (Figure 1B) indicating expression of Chrm1. Anti-GFP immunostained image of the sagittal section of adult Chrm1 BAC transgenic mouse brain also indicates Chrm1 expression in the cerebral cortex and hippocampus regions (Figure 1C). Furthermore, RNA *in situ* hybridization data obtained from the St. Jude Brain Gene Expression Map (BGEM) database (Magdaleno et al., 2006) generated by hybridizing RNA probes specific to the 3′ untranslated region of Chrm1 mRNA, revealed Chrm1 expression in the adult mouse cerebral cortex and hippocampus (Figure 1D, blue and yellow arrows, respectively). Expression of Chrm1 in mouse cortex and hippocampus matched our reporting of CHRM1 expression in the same brain regions in humans and pigs (Sabbir et al., 2022) indicating a conserved pattern of expression across mammalian species. This encouraged us to study Chrm1^-/-^ mouse cortical tissue-derived mitochondria as a model system to understand the pathophysiological mechanism involved in the reported poor survival of a subset of AD patients with severe loss of cortical CHRM1 compared to age-matched unaffected individuals (Sabbir et al., 2022).

### 3.2 Immunoblotting and TEM-based analysis revealed isolated cortical mitochondrial fractions were enriched in Chrm1 proteins and synaptic terminals containing intact mitochondria

The theoretical molecular weight of mouse Chrm1 protein (Transcript ID: ENSMUST00000035444.9) is 51 kDa (kDa). Previously, using an extensively validated anti-Chrm1 antibody, we demonstrated that post-translational modifications (PTMs: glycosylation), cause the appearance of 70-75 kDa forms of CHRM1 in cortex and hippocampus tissues derived from human and pig brains (Sabbir et al., 2022). Immunoblotting using enriched mitochondrial fractions as well as whole tissue lysates derived from wild-type cortex and hippocampus revealed the presence of 51 and 72 kDa anti-Chrm1 immune-reactive bands in both tissues (Figure 1E). In contrast, the enriched mitochondrial fractions derived from both tissues displayed only the 72 kDa band indicating PTMs of Chrm1 are responsible for its intracellular localization (Figure 1E). Furthermore, the p72 anti-Chrm1 bands in the mouse cortex and hippocampus-based immunoblots appeared compact which is in contrast to the diffused p70-75 anti-CHRM1 immunoreactive bands previously observed in *postmortem* human cortex and hippocampus-based immunoblots (Sabbir et al., 2022). Interestingly, compact Chrm1 immunoreactive bands have been observed in the mouse brain (striatum) (Lee et al., 2013) and skin (Uberti et al., 2018) tissue using similar antibodies from Santa Cruz Biotechnology. The discrepancy between the appearance of anti-Chrm1 bands in human versus mouse tissue-based immunoblots may be due to cross-species differences. We used Voltage Dependent Anion Channel (Vdac1), an outer mitochondrial membrane-localized protein, to quantify Chrm1 enrichment in the mitochondrial fractions (Figure 1E). Quantification based on immunoblotting revealed significant (*p* < 0.0001, by multiple comparisons, one-way ANOVA test) enrichment (2-3-fold) of both Vdac1 and Chrm1 proteins in the enriched mitochondrial fractions compared to cortical and hippocampal tissue lysates (Figure 1F). Next, we performed TEM analysis to characterize the enrichment of synaptic mitochondria in this fraction. TEM analysis of the enriched mitochondrial fractions (Figure 1G) revealed the presence of both intact isolated mitochondria (Figure 1H, white arrow) as well as membrane-bound mitochondria (Figure 1H,I, yellow arrows) present in the excitatory presynaptic dendritic terminals (Figure 1H,I, pink arrows). The excitatory presynaptic terminals in the isolated cortical fractions were identified by the presence of synaptic vesicles (Figure 1H,I, red arrows) adjacent to the synaptic cleft (Figure 1H,I, blue arrows). The postsynaptic terminals (Figure 1H,I, green arrows) were characterized by relatively less abundance of synaptic vesicles compared to presynaptic terminals and were less electron-dense (contrasted). Furthermore, we observed loss of cristae in the presynaptic mitochondria derived from Chrm1^-/-^ cortex compared to wild-type (Figure 1H,I, yellow arrows). This phenotype has been discussed in detail in section 3.9. Overall findings indicate that our enriched cortical mitochondrial fractions contained dendritic synaptic terminals with intact mitochondria and exhibited abundant post-translationally modified Chrm1 proteins.

### 3.3 Deletion of Chrm1 dampened functioning of complexes II-IV mediated respiration and the maximal respiratory capacity in mouse cortical mitochondria under a coupled state

**FIGURE 2:**
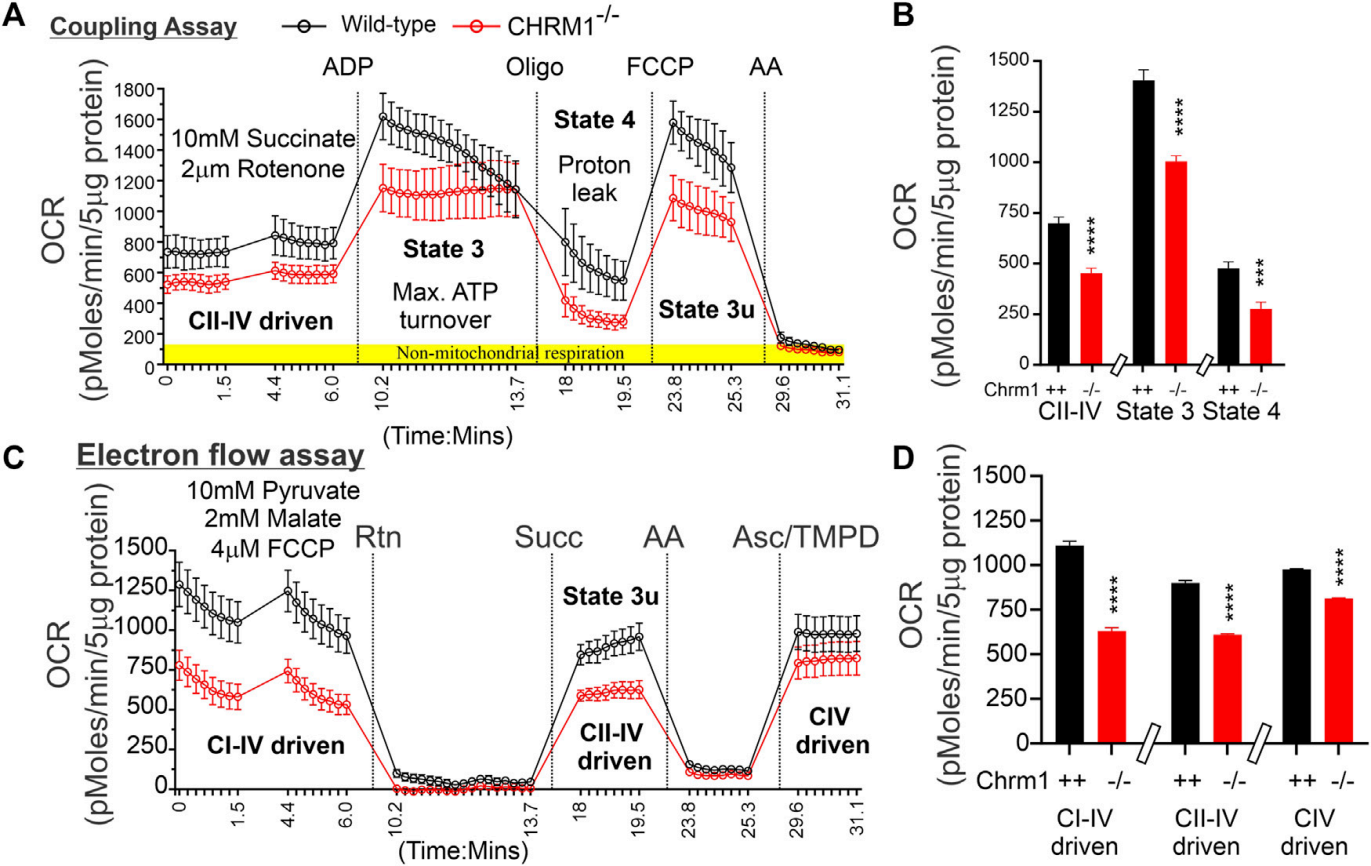
Coupling and electron flow assay using isolated cortical mitochondria. **(A and C)** Line graphs showing OCR kinetics in coupling **(A)** and electron flow **(C)** assays performed simultaneously in a Seahorse 24X flux analyzer using 5 µg protein equivalent of enriched cortical mitochondria derived from adult wild-type and Chrm1^-/-^ mice. The final concentration of the inhibitors and substrates is mentioned in the text. The data were generated using a “point-to-point” mode in the Seahorse XF24 software package. The point-to-point displays OCR as a series of rates across the measurement period and can show changes in the rate across the measurement period. The OCR kinetics data presented in **(A and C)** was converted to a “middle point” mode using Seahorse 24X flux analyzer software utility which is a preferred method for statistical comparison between groups (wild-type and chrm1^−/−^). The middle point mode shows a single OCR value for the measurement period which is the average of the point-to-point rates. **(B)** Bar graphs showing complexes I-IV (coupled), State 3 (coupled), and state 4 (coupled) respiration (OCR). The non-mitochondrial respiration was subtracted to calculate the basal respiration at different states. *N* = 20 replicates from 2 independent experiments. Data represented as Mean ± SEM. Comparison of the means involving wild-type and Chrm1^-/-^ groups in this dataset and subsequent datasets were by *t*-test (unpaired). **(D)** Bar graphs showing complexes I-IV, complexes II-IV, and complex IV-mediated uncoupled respiration. Data represented as Mean ± SEM.

We performed coupling and electron flow assays (Sabbir et al., 2021) to assess isolated cortical mitochondrial function in wild-type and Chrm1^−/−^ mice. The fundamental working principles of these assays have been previously described in detail by Sabbir et al. (2021). The coupling assay measures the level of respiratory coupling between the electron transport chain (ETC.) and the oxidative phosphorylation OXPHOS machinery and is reflected by the increase in respiration rate in response to ADP (Figure 2A). Coupling assay revealed that in the presence of succinate and rotenone (complex I inhibitor), complexes II-IV driven basal respiration was significantly (*p* < 0.0001) decreased in Chrm1^-/-^ cortical mitochondrial fractions compared to wild-type (Figure 2B). The ADP-stimulated State 3 respiration was also significantly (*p* < 0.0001) low in Chrm1^-/-^ cortical mitochondria compared to wild-type (Figure 2A,B). Similarly, there was a significantly (*p* = 0.0009) decreased proton leak (state 4; dissipation of protons in the presence of ATP synthase inhibitor Oligomycin) in Chrm1^-/-^ cortical mitochondria compared to wild-type (Figure 2A,B). The proton leak is usually measured indirectly by measuring the mitochondrial oxygen consumption under non-phosphorylating conditions, that is in the presence of oligomycin (Porter et al., 1999; Jastroch et al., 2010). We measured the coupling efficiency (the percentage of respiration rate at a given mitochondrial membrane potential that is used for ATP synthesis) by calculating the respiratory control ratio (RCR: State 3/State 4) (Sabbir et al., 2021). The RCR was significantly (*p* < 0.0001, unpaired *t*-test) increased in the Chrm1^-/-^ cortical mitochondrial fractions compared to wild-type (Supplementary Figure S1A). A high RCR means efficient and healthy mitochondrial functioning because it implies that the mitochondria have a high capacity for substrate oxidation and a low proton leak (Brand and Nicholls, 2011). We anticipated that Chrm1^-/-^ cortical mitochondria would exhibit a low RCR compared to the wild-type because the state 3 respiration was low under the Chrm1 loss condition compared to the wild-type, but the decreased proton leak in the Chrm1^-/-^ cortical mitochondria caused an apparent increase in the RCR which, in this case, is not reflective of the true mitochondrial functionality. In this context, it is important to note that there is no absolute RCR value that is diagnostic of dysfunctional mitochondria, because values depend on numerous factors, a change in almost any aspect of oxidative phosphorylation will change RCR (Brand and Nicholls, 2011). Overall, the coupling assay revealed significantly decreased cortical mitochondrial OXPHOS functioning under Chrm1 loss of function condition.

### 3.4 Deletion of Chrm1 significantly decreased complex-I and II-driven uncoupled (plus FCCP) oxygen consumption in isolated cortical mitochondria

The electron flow experiment is designed to follow and interrogate each complex of the electron transport system under an uncoupled state by the addition of FCCP (Figure 2C) (Sabbir et al., 2021). The complexes I-IV, complexes II-IV, and complex IV-mediated respiration under uncoupled state were found significantly (*p* < 0.0001, unpaired *t*-test) low in isolated cortical-derived enriched mitochondria compared to wild-type (Figure 2D). The biological relevance of respiration under the FCCP-induced uncoupled state is not known (Sabbir et al., 2021), however, overall findings indicate some difference in OXPHOS functioning in the cortical mitochondria under Chrm1 loss of function condition. We examined the relative abundance of Ndufb8 (complex I), Sdhb (complex II), Uqcrc2 (complex III), Mtco1 (complex IV), and Atp5a (complex V) by WB to understand if a loss of these protein subunits was underlying the functional deficits in Chrm1^-/-^ cortical mitochondrial (Supplementary Figure S1B). Quantification based on immunoblotting revealed no statistical difference in the relative amount of these proteins under Chrm1 loss condition (Supplementary Figure S1C).

### 3.5 Deletion of Chrm1 altered the ultrastructural organization of ATP synthase and respiratory complexes in the cortical mitochondria

**FIGURE 3:**
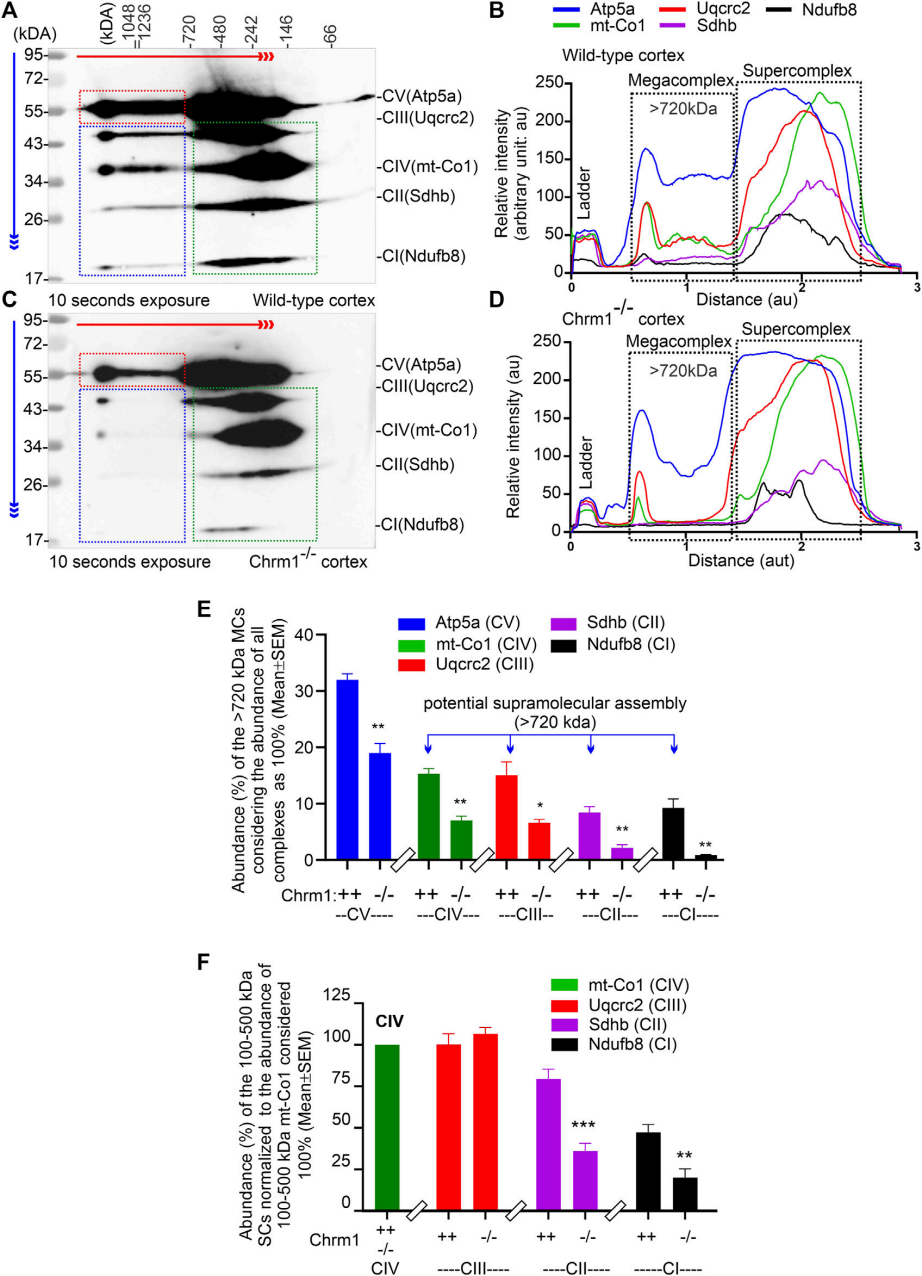
2D BN-PAGE/SD-PAGE analysis revealed that loss of Chrm1 led to the abnormal supramolecular assembly of respiratory protein complexes in cortical mitochondria. **(A and C)** Immunoblots showing OXPHOS-associated MPCs in wild-type **(A)** and Chrm1^−/−^ **(C)** enriched cortical mitochondria. The immunoblot was generated by the simultaneous use of a cocktail of five antibodies ​(Table 1). The red and blue arrows indicate the direction of electrophoresis during 1D BN-PAGE and 2D SDS-PAGE respectively. The red dotted rectangle indicates ≥720 kDa Atp5a complexes representing ATP synthase oligomerization. The blue and green dotted rectangles show vertical alignments of C1-IV associated protein subunits indicating potential MCs (blue) and SCs (green). Relatively lower exposure images of the immunoblots presented in A and C are provided in Supplementary Figure S2 to highlight bands that may appear to be oversaturated. All quantification was based on suboptimal exposure of immunoblots for the respective proteins to avoid oversaturation of the band images. **(B and D)** Image J-based plot profile of the immunoblots presented in **(A and C)**. Black dotted rectangles indicate the relative abundance of the ≥720 kDa MCs and 100-500 kDa SCs. **(E and F)** Bar graphs showing the relative proportion of complexes I-V protein subunits associated with ≥720 kDa MCs **(E)** or 100-500 kDa SCs **(F)**. Data presented as Mean ± SEM. *N* = 3 independent experiments using 3 mice. The bar graph in F shows the relative abundance of Uqcrc2 (complex III), Sdhb (complex II), and Nsufb8 (complex I) subunits aligned vertically with mt-Co1 (complex IV) subunit in 100-500 kDa SCs. The abundance of Uqcrc2/Sdhb/Ndufb8 proteins in the 100-500 kDa region in the immunoblot **(A and C)**, (green dotted rectangles) was normalized relative to the abundance of mt-Co1 which was considered as 100%. Data presented as Mean ± SEM. *N* = 3 independent experiments using 3 mice. In all datasets, the means of wild-type and Chrm1^-/-^ groups were compared by *t*-test (unpaired).

The respiratory MC/SC represents the highest-order assembly of OXPHOS complexes allowing mitochondria to respond to energy-requiring conditions. Therefore, to understand the molecular basis of the abnormal mitochondrial function under Chrm1 deletion condition, we studied respiratory MC/SC ultrastructure using a cocktail of anti-Ndufb8/Sdhb/Uqcrc2/mt-Co1/Atp5a antibodies to simultaneously detect complexes I-V-associated MPCs separated by 2D BN-PAGE/SDS-PAGE. The supramolecular assembly of the OXPHOS MPCs exhibited a considerable difference between Chmr1^-/-^ and wild-type cortical mitochondria (Figure 3A–D; Supplementary Figure S2A–H). The Atp5a (complex V) appeared as dimeric to oligomeric complexes ranging from ∼100 kDa to ≥1 Megadalton (MDa) MPCs (Figure 3A,C; Supplementary Figure S2A–D). Quantification based on immunoblots (below saturation levels during the chemiluminescent detection of the respective proteins) and plot profiles (Supplementary Figure S2A–H) of the protein complexes revealed significantly decreased ≥720 kDa Atp5a oligomers in Chmr1^-/-^ cortical mitochondria compared to wild-type (Figure 3E). In transformed human cells, the molecular weight of respiratory chain MC complexes (complex I_1_III_2_IV_1_) has been reported as ≥1.7 MDa (Guo et al., 2017a). False-colored overlapped immunoblots of the OXPHOS MPCs profile revealed vertical alignment of Ndufb8, Sdhb, Uqcrc2, and mt-Co2 oligomers in the ≥720 kDa and 100-500 kDa regions representing potential MC and SC structures (Figure 3B,D; Supplementary Figure S2E–H) respectively. Based on existing reports in the literature (Acín-Pérez et al., 2008), we considered ≥720 kDa and 100-500 kDa MPCs as MCs and SCs (Figure 3B,D, black dotted rectangles), respectively. Quantification based on immunoblots (below saturation levels during the chemiluminescent detection of the respective proteins) and plot profiles of the protein complexes revealed significantly low ≥720 kDa complexes I-IV oligomers (MCs) formed in Chmr1^-/-^ cortical mitochondria compared to wild-type (Figure 3E). On the other hand, the appearance of the 100-500 kDa Uqcrc2, mt-Co1, Sdhb, and Ndufb8-associated SCs was also affected under Chrm1 deletion condition (Figure 3B,D, black dotted rectangles). Relative quantification (below saturation levels during the chemiluminescent detection of the respective proteins) based on immunoblots and plot profiles of these MPCs (normalized to 100-500 kDa mt-Co1 level considered as 100%) revealed a significantly decreased amount of Ndufb8 (complex I) and Sdhb (complex II) oligomers vertically aligned with the Uqcrc2 (complex III)/mt-Co1(complex IV) oligomers in the 100-500 kDa region indicating a potential disruption in SC structures in Chmr1^-/-^ cortical mitochondria compared to wild-type (Figure 3F). Overall findings indicate that deletion of Chrm1 led to a dramatic change in the ultrastructural organization of ATP Synthase (Atp5a) and complexes I-IV-associated MPCs which may underlie the observed functional deficits in the isolated cortical mitochondria.

**TABLE 1:** List of antibodies.

Antibody	Catalog number	Vendor	Type	Lot number
Chrm1	Sc-365966 (G-9)	Santa Cruz Biotechnology	Mouse monoclonal	F2812
GFP	Sc-9996	Santa Cruz Biotechnology	Mouse monoclonal	G1615
OXPHOS-rodent	MS604-300	Abcam	Mouse monoclonal	K2342
Vdac1	Sc-390996	Santa Cruz Biotechnology	Mouse monoclonal	D1918

### 3.6 Overexpression of Chrm1 in mouse tumor cells that do not express native Chrm1 improved ATP synthase ultrastructure and mitochondrial function

**FIGURE 4:**
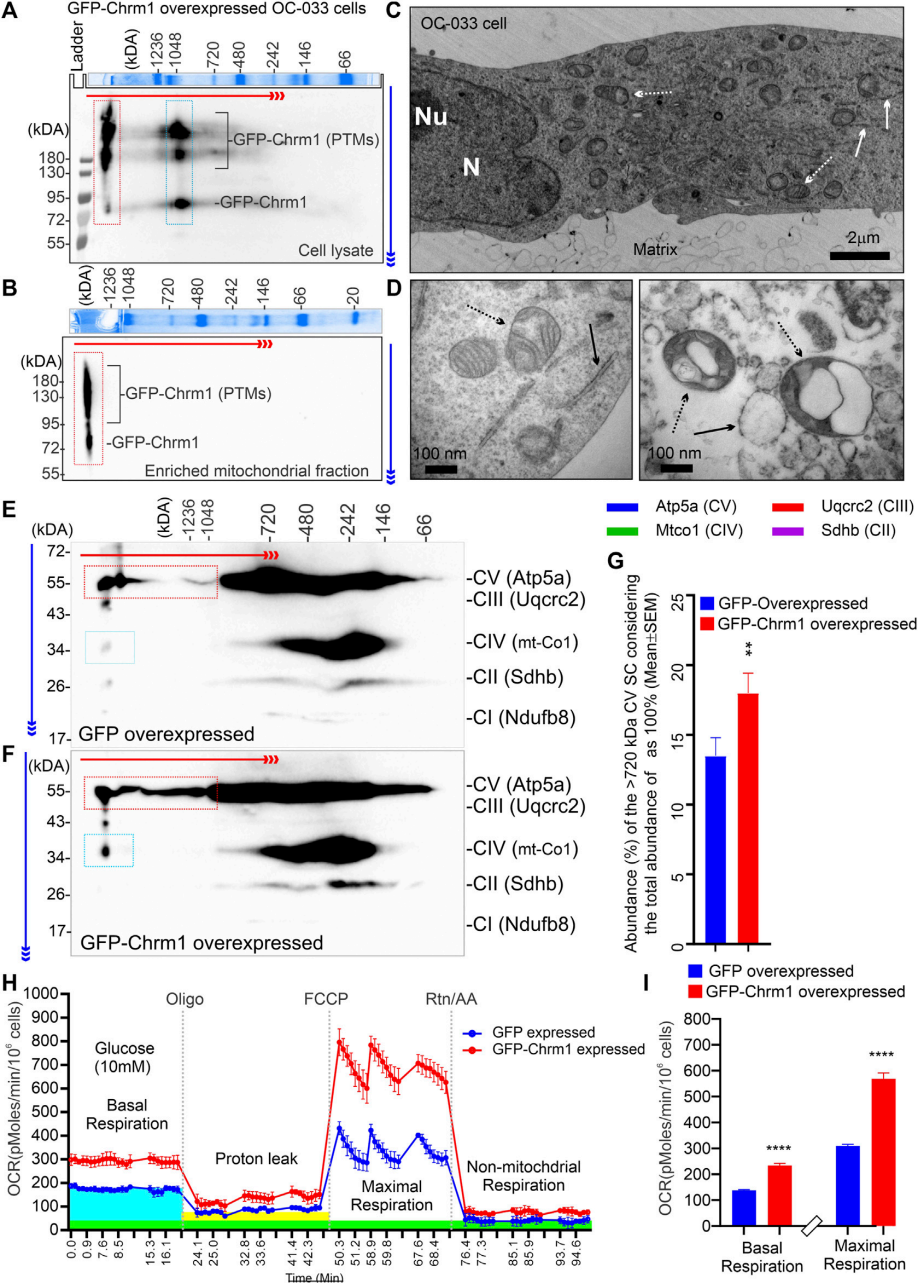
Effect of N-terminal GFP-tagged Chrm1 overexpression on OXPHOS supramolecular assembly in OC-033 ovarian tumor cells that do not express native Chrm1. **(A and B)** Immunoblots showing Chrm1-associated protein complexes in GFP-Chrm1 overexpressed OC-033 tumor cells **(A)** and enriched mitochondrial fractions derived from the overexpressed cells **(B)**. The proteins were separated by 2D BN-PAGE/SDS-PAGE. The Coomassie-stained gel slice at the top panels shows first dimension separation of native page molecular weight markers. The red and blue arrows indicate the direction of BN-PAGE and SDS-PAGE, respectively. The red rectangle area indicates ≥1200 kDa Chrm1-MPCs present the enriched mitochondrial fraction. The blue rectangle area represents 1000 kDa Chrm1-MPCs not coenriched with the ER/mitochondrial fractions. **(C–D)** TEM images showing the ultrastructure of the OC-033 cell grown on a nitrocellulose membrane (matrix) **(C)** and the enriched mitochondrial fraction derived from OC-033 cells **(D)**. Solid white/black arrows: rough ERs, white/black dotted arrows: mitochondria, N: Nucleus, and Nu: Nucleolus. **(E–F)** Immunoblots showing OXPHOS protein complexes in GFP **(E)** and GFP-Chrm1 **(F)** overexpressed enriched mitochondrial fractions derived from transiently transfected OC-033 cells. The red and blue rectangles indicate the relative abundance of Atp5a (complex V) and mt-Co1 (complex IV) associated MCs in mitochondrial fractions derived from Chrm1-GFP/GFP overexpressed cells. **(G)** Bar graph showing the relative proportion of ≥1000 kDa complex VMCs. *N* = 3 independent experiments. The *p*-value was calculated by *t*-test (unpaired). **(H)** Cellular respiration in GFP and GFP-Chrm1 overexpressed OC-033 cells was measured using an XF-24 extracellular flux analyzer. Line graphs showing OCR kinetics at different time points following injection of Oligo (Oligomycin), FCCP (Carbonyl cyanide 4-trifluoromethoxy), and Rtn/AA (rotenone/antimycin). The blue, green, and yellow highlighted areas indicate basal respiration following the injection of glucose, non-mitochondrial respiration following the Rtn/AA injection, and proton leak following the injection of Oligomycin, respectively. Data presented as Mean ± SEM, *N* = 10 replicates. **(I)** Bar graphs show basal respiration and maximal respiratory capacity of the GFP and GFP-Chrm1 overexpressed cells. Basal respiration = (last rate measurement before oligomycin injection)—(non-mitochondrial respiration rate). Maximal respiration = (maximum rate measurement after FCCP injection)—(non-mitochondrial respiration). Data presented as Mean ± SEM, *N* = 20 replicates from 2 independent experiments. Statistical significance by *t*-test (unpaired).

We overexpressed GFP-M1 in oncogenic K-RasG12D transformed ovarian cancer-derived mouse tumor cells (OC-033) developed by Sabbir et al. (2012) and studied OXPHOS architecture and function. 2D BN-PAGE/SDS-PAGE analysis using total cellular lysates from GFP-Chrm1 expressed OC-033 cells revealed that Chrm1 formed 2 major protein complexes at ≥1200 and –1000 kDa, respectively (Figure 4A, red and blue dotted rectangles, respectively). The theoretical molecular weight of the recombinant GFP-Chrm1 is 78 kDa, but potential PTMs caused the appearance of ≥130 kDa forms (Figure 4A). BN-PAGE analysis of the enriched mitochondrial fractions revealed that only the ≥1200 kDa Chrm1 complex was present in the enriched mitochondrial fraction (Figure 4B, red dotted rectangle). TEM examination of the enriched mitochondrial fraction revealed the presence of rough endoplasmic reticulum (RER) like structures as well as isolated mitochondria in the enriched mitochondrial fractions (Figures 4C,D). BN-PAGE analysis revealed that the expression of Chrm1 in OC-033 cells significantly increased the abundance of ≥1000 kDa Atp5a oligomers compared to GFP-expressed cells (Figures 4E–G; Supplementary Figure S1A and B). Furthermore, there is a relative increase in the appearance of ≥1000 kDa mt-Co1 oligomers in GFP-Chrm1 expressed cells compared to GFP expressed cells (Figure 4E,F, blue rectangles, Supplementary Figure S3A,B) indicating potential improvement in MC formation in the presence of Chrm1. Overall findings indicate that expression of Chrm1 in OC-033 cells altered mitochondrial OXPHOS assembly, specifically ATP synthase (Atp5a) oligomerization, and MC formation. Next, we performed mitochondrial functional analysis (Sabbir, 2019) using GFP-Chrm1 and GFP overexpressed OC-033 cells (Supplementary Figure S3C,D) to see if improved OXPHOS architecture in Chrm1 overexpressed cells has functional consequences (Figure 4H). The basal respiration (OCR) and the maximum respiratory capacity were significantly high in GFP-Chrm1 overexpressed cells compared to GFP overexpressed cells (Figure 4I). These findings indicate that Chrm1 overexpression led to an improved ATP synthase oligomerization and MC assembly leading to mitochondrial functional improvement.

### 3.8 Deletion of Chrm1 significantly increased the proportion of hyperchromic (dark) neurons with abnormal mitochondria and ER in the cerebral cortex

**FIGURE 5:**
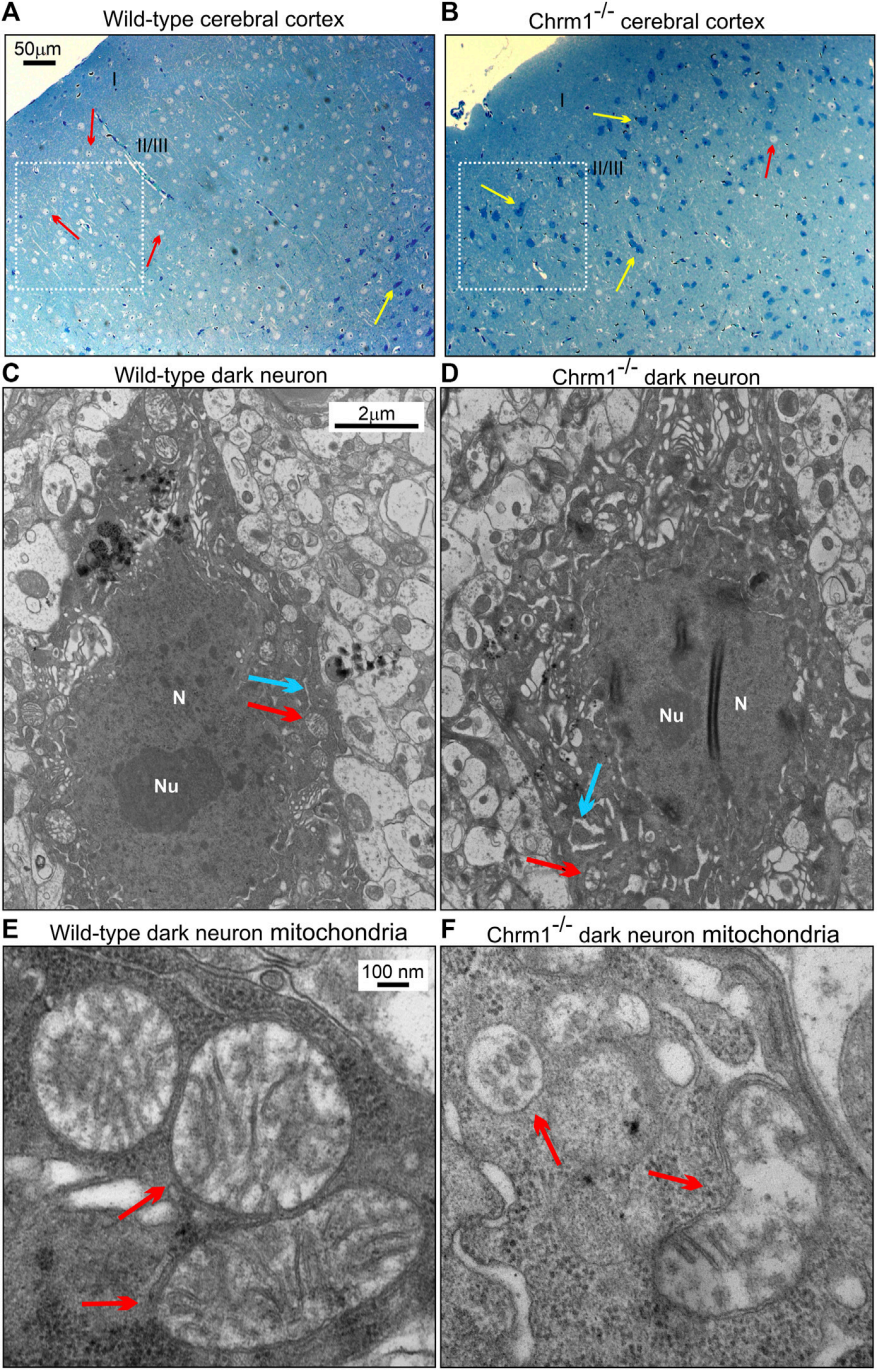
Chrm1 loss affected the distribution of dark pyramidal neurons in the cerebral cortex. **(A–B)** Toluidine-stained ultrathin sections of the cerebral cortex somatosensory region II/III (Belgard et al., 2011) (white dotted rectangles) in the wild-type **(A)** and Chrm1^-/-^ **(B)** mice. The red arrow is a light neuron and the yellow arrow is a dark neuron. **(C–D)** TEM images showing the ultrastructure of dark neurons in the II/III somatosensory cortex. The red arrow: normal/abnormal mitochondrion. The blue arrow: normal/swelled cisternae of the ER. N: Nucleus. Note abnormally distended cisternae of the rough ER in the Chrm1^-/-^ cortical dark neuron compared to wild-type **(C)**. **(E–F)** TEM images at high magnification showing the ultrastructure of the wild-type and Chrm1^-/-^ cortical dark neuron mitochondria. The red arrows are normal/abnormal mitochondria.

We studied the distribution of cortical pyramidal neurons by ultrathin sectioning of the cerebral cortex followed by toluidine staining to understand the impact of Chrm1 loss on neuronal phenotype and distribution. The wild-type and Chrm1^-/-^ cortical tissue samples were processed in parallel. Toluidine-stained semithin sections (–500 nm thick) of the wild-type cortex revealed the abundance of pyramidal (light/hypochromic neurons) neurons (Figure 5A, red arrows) in different cortical layers, including the somatosensory layer II/III (Figure 5A, white dotted rectangle). In addition, we observed a few dark (hyperchromatic) neurons (Figure 5A, yellow arrow) mainly in the deeper cortical region (Figure 5A) in wild-type mice. In contrast, the Chrm1^-/-^ cortex exhibited an abundance of dark neurons in all cortical regions (Figure 5B, white dotted rectangle, yellow arrows) corresponding to a decrease in the hypochromic pyramidal neurons (Figure 5B, red arrow). Dark neurons were previously observed in optical and electron microscopic analysis of the cortex (Murakami et al., 1997) and the frequency of dark neurons in adult rat frontal cortex has been reported as 2% (Zimatkin and Bon’, 2018). Manual counting of the light and dark neurons in the ultrathin cortical sections (12 sections at ×20 magnification from different parts of the cortex, *N* = 3 mice) revealed 82.17% ± 1.9% (Mean ± SE) of the Chrm1^-/-^ cortical neurons are dark compared to 5.4% ± 0.6% (Mean ± SE) in wild-type. The significant (*p* < 0.0001 by *t*-test) increase of the dark neurons in the Chrm1^-/-^ cortex indicates neurophysiological changes. We performed a TEM analysis of the dark neurons to unravel any structural aberration under Chrm1 loss condition (Figures 5C–E; Supplementary Figure S4A–F). The dark neurons in Chrm1^-/-^ cortex exhibited abnormal mitochondria with a lack of well-organized cristate compared to wild-type (Figure 5E,F, red arrows). In addition, the dark neurons in Chrm1^-/-^ cortex exhibited ERs with heavily dilated cisterns compared to wild-type (Figure 5C,D, blue arrows; Supplementary Figure S4C–F).

### 3.9 Deletion of Chrm1 severely affected mitochondrial structure and physiochemical properties of the synapses in the cortical neuropil

**FIGURE 6:**
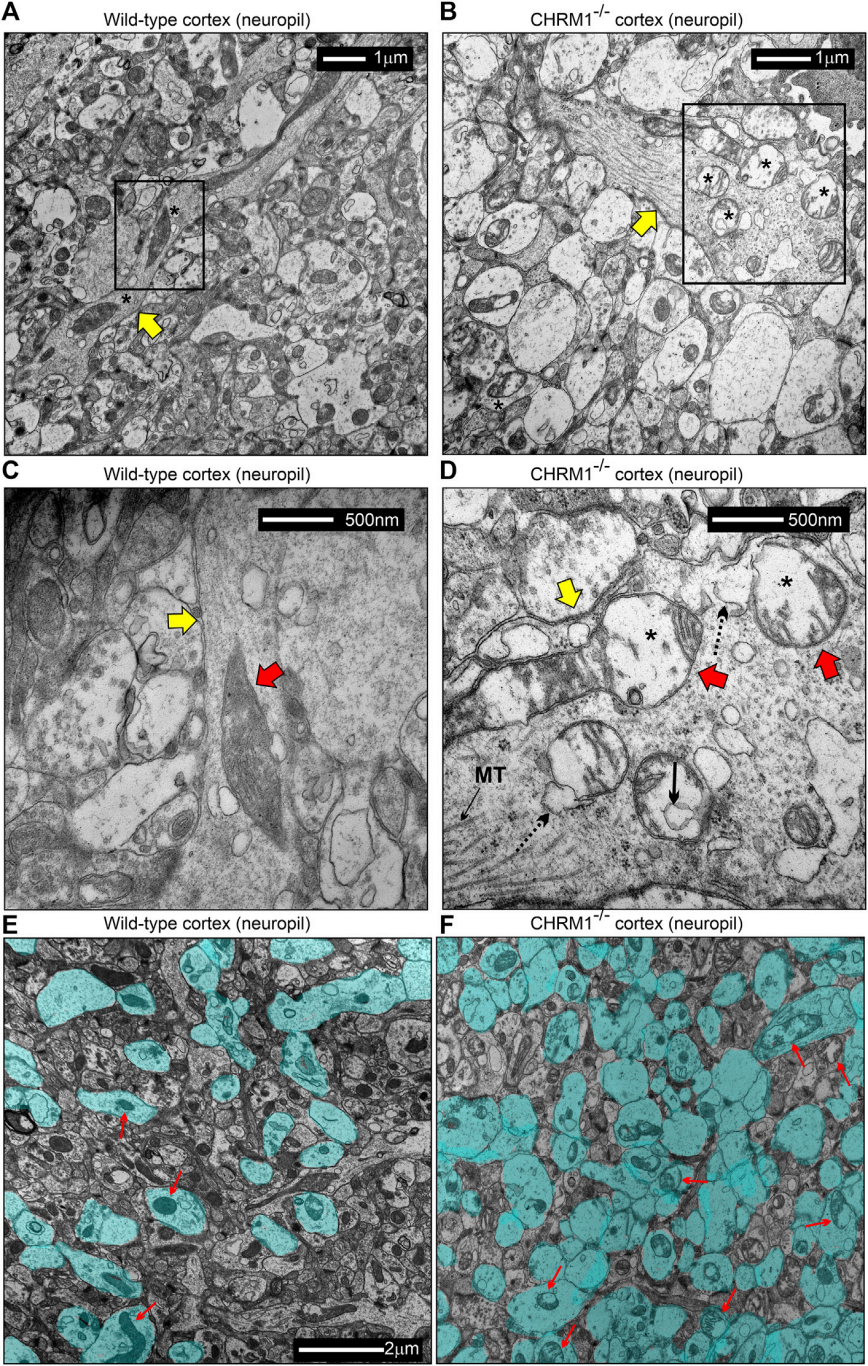
Mitochondrial morphology is deranged in the neuropil of the cerebral cortex in Chrm1^-/-^ mice. **(A–B)** TEM images showing the ultrastructure of mitochondria in the II/III somatosensory cortical neuropil in wild-type **(A)** and Chrm1^-/-^ **(B)** mice. The black rectangles represent the area that has been shown in a magnified view in the corresponding images in **(C)** and **(D)**. The Yellow arrows are dendritic processes containing normal/abnormal (red arrows) mitochondria. Asterisk: empty (less electron-dense) matrix. **(C and D)** TEM images showing the presence of normal/abnormal mitochondria (red arrows) in the dendritic process (yellow arrow) in the cortical neuropil of wild-type and Chrm1^-/-^ mouse respectively. See the text for a detailed description of the abnormalities. Black dotted arrow: abnormally swollen outer mitochondrial membrane, black solid arrow: a potential influx of cytoplasmic contents into the mitochondria, black asterisks: empty matrix content. **(E and F)** TEM images showing the tinctorial (double contrast staining with uranyl acetate and lead citrate) property and mitochondrial abnormalities in the synaptic terminals in cortical neuropil in wild-type **(E)** and Chrm1^-/-^ **(F)** mice. The neuropil contained both “less contrasted” and “high contrasted” synaptic terminals. The less contrasted synapses were falsely colored (blue) to quantify (Supplementary Figure S3) and highlight their differential abundance. Red arrows indicate synapses containing normal/abnormal mitochondria.

**FIGURE 7:**
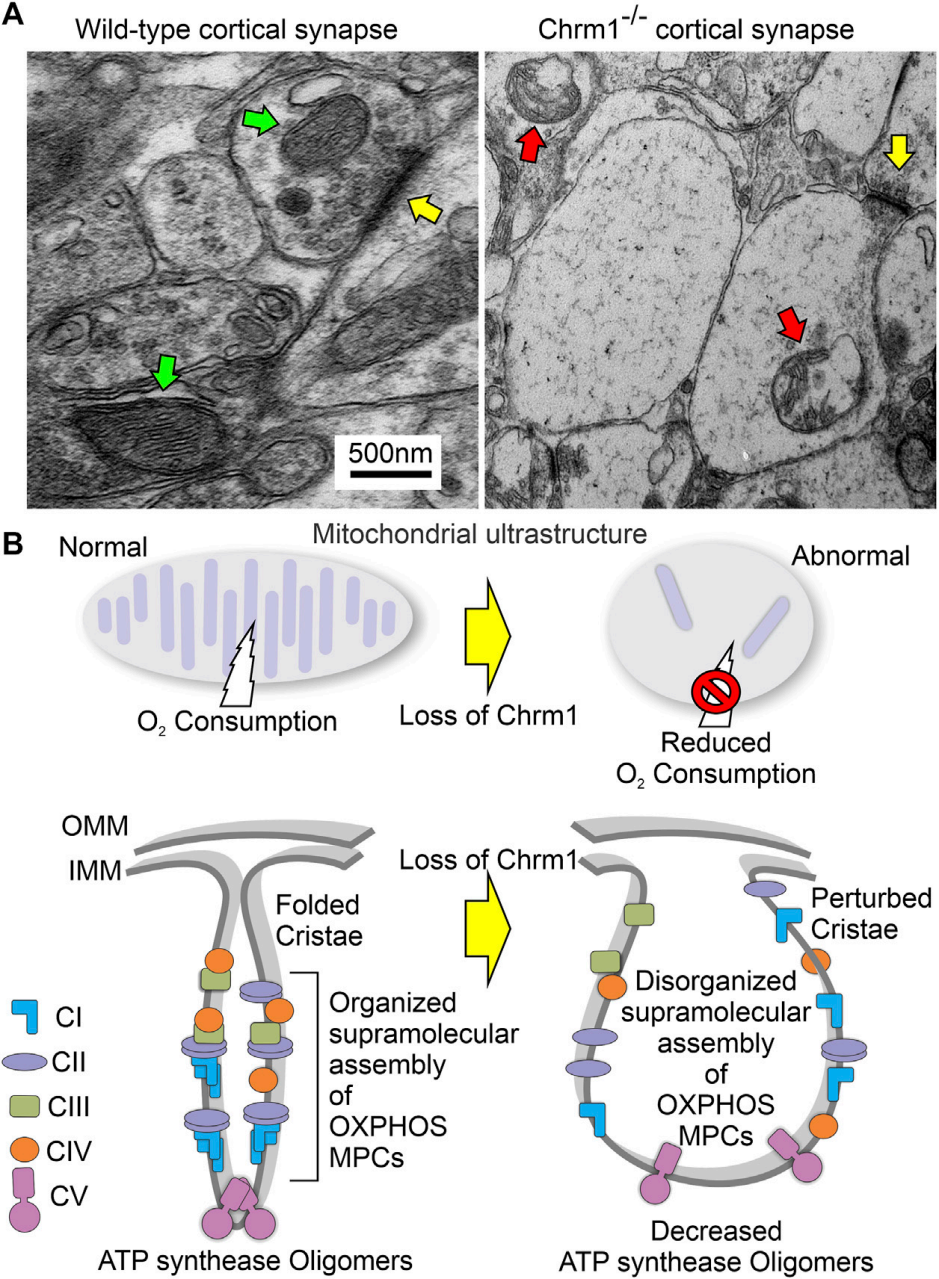
Model showing the ultrastructural organization of cristae and the overall effect of loss of Chrm1 in mouse cortex. **(A)** TEM images of the presynaptic dendritic ends in Chrm1^-/-^ and wild-type cortical neuropil highlighting mitochondrial abnormalities. Yellow arrows: synapse, red arrows: abnormal mitochondria, and green arrows: normal mitochondria. **(B)** Model showing the impact of the Chrm1 loss on cristae shape, ATP synthase oligomers, respiratory SC/MC structures, and mitochondrial function, all interdependent factors. Cristae shape depends on the proper assembly and stabilization of respiratory SCs and MCs. The proteins involved in the maintenance of cristae structure are also involved in SC formation. In this model, a portion of the outer mitochondrial membrane (OMM), inner mitochondrial membrane (IMM), and cristae is shown.

TEM-based analysis of the cortical neuropil revealed the presence of abnormal mitochondria in the Chrm1^-/-^ dendritic processes (Figures 6A–D, red arrows) and also in the synapses (Figure 6E,F, red arrows) compared to wild-type. Mitochondria in the Chrm1^-/-^ cortex were swollen with severe loss of cristae (red arrows), effacement of cristae (black dotted/solid arrow), disruption of mitochondrial membranes, and exhibited empty (hypochromic, asterisks) matrix (Figures 6B,D,F) compared to wild-type (Figures 6A,C,E). In addition, we observed visual differences in the contrasting property of the dendritic synapses in the TEM images of cortical neuropil. The contrasting property is based on the use of heavy metals uranium and lead, in the form of uranyl acetate and lead citrate that reacts with the organic matter and provides contrast because of the differences in the electron density of the stained organic molecules in the cell. Darker areas in the TEM image indicate that the organic content is denser in that sample area. We observed a significantly (*p* < 0.0001) increased proportion of less electron-dense (blue highlighted) synaptic terminals mostly containing abnormal mitochondria (red arrows) in Chrm1^-/-^ cortical neuropil compared to wild-type (Figure 6E,F; Supplementary Figure S5A). Furthermore, the cortical synaptic terminals exhibited a significantly (*p* < 0.0001) higher proportion of abnormal mitochondria (empty matrix, membrane protrusion, disorganized cristate) in the Chrm1^-/-^ neuropil compared to wild-type (Figure 6E,F; Supplementary Figure S5B). Overall, the TEM-based study revealed a considerable difference in the tinctorial property of the synaptic nerve terminals under Chrm1^-/-^ loss condition which is possibly due to a change in the physiochemical property of the nerve endings (Figure 7A). Furthermore, Chrm1 loss compromised both pre-and post-synaptic dendritic mitochondrial ultrastructural integrity (Figures 7A,B).

## 4 Discussion

In this study, we report for the first time that loss of Chrm1 affected the supramolecular assembly of OXPHOS protein complexes, impaired mitochondria ultrastructure, and reduced respiration in adult cortical neurons. Our study indicates that Chrm1 loss led to an altered ATP synthase (Atp5a) oligomerization and complex 1-IV respirasome MC/SC assembly (Figure 3), all underlying the structural and functional deficits in the Chrm1^-/-^ cortical mitochondria compared to wild-type. Overexpression of Chrm1 in transformed cells not expressing native Chrm1 led to the improvement of ATP synthase (Atp5a) oligomerization, respirasome assembly, and cellular respiration, providing further support for Chrm1-mediated regulation mitochondrial physiology. We also demonstrated that Chrm1 loss significantly altered the physiochemical nature (tinctorial property) of cortical neurons and synapses. The significance of this study is that it has uncovered a previously unknown cause-and-effect relationship between Chrm1 signaling loss and reduced mitochondrial respiration. This points to the molecular and structural abnormalities in the mitochondria observed under Chrm1 loss of function condition that were not reported previously. These findings provide novel insight into cholinergic hypofunction and mitochondrial dysfunction in the cortical neurons potentially underlying the pathogenesis of Alzheimer’s (Jiang et al., 2014).

One of the major findings is significantly reduced ≥720 kDa Atp5a oligomers in the cortical mitochondria under Chrm1 loss condition associated with abnormal mitochondrial structure and function (Figure 3). Overexpression of Chrm1 in transformed OC-033 cells significantly increased Atp5a oligomerization which was associated with significantly increased basal respiration as well as enhancement in maximal uncoupled respiratory capacity (Figure 4). Atp5a is a component of the mitochondrial ATP synthase enzyme complex catalyzing ATP synthesis, using an electrochemical gradient of protons across the inner membrane during oxidative phosphorylation. ATP synthase is composed of two linked multi-subunit complexes: the soluble catalytic core (F1) and the membrane-spanning proton channel component (Fo) (Jonckheere et al., 2012). The F1 catalytic core consists of 5 different subunits, three α (ATP5F1A), three β (ATP5F1B), and one γ (ATP5F1C), δ (ATP5F1D) and ε (ATP5F1E) (Jonckheere et al., 2012). Cryoelectron microscopy revealed that ATP synthase forms a dimer, and the dimer is sharply bent to help shape the extensively folded inner membrane of the mitochondrion (Guo et al., 2017b). Furthermore, dimer pairs from tetramers which in turn oligomerize in long rows, forming ATP synthase complexes that force the membrane to maintain its convexity at the apex of cristae (Nesci and Pagliarani, 2019). In our BN-PAGE/SDS-PAGE analysis, the appearance of high molecular weight Atp5a complexes (≥100 kDa, Figure 3) matched an identical profile of multimeric ATP synthase oligomers in digitonin soluble fractions of rat heart mitochondria separated by a similar technique involving first dimension native electrophoresis and second dimension SDS-PAGE (Wittig et al., 2006). Therefore, it is reasonable to suggest that the abnormal ATP synthase oligomerization represented by the significant loss of ≥720 kDa Atp5a oligomers in Chrm1^-/-^ cortical mitochondria may be responsible for the defective cristae structure underlying dampened mitochondrial function.

Another major finding is that the abnormal supramolecular assembly of OXPHOS protein complexes revealed by native BN-PAGE analysis was associated with significantly reduced OXPHOS function in Chrm1^-/-^ cortical mitochondria under both coupled (no FCCP) and uncoupled (presence of FCCP) states. The modern concept of mitochondrial architecture states that the organization of the components of the OXPHOS system changes from freely moving to super-assembled structures, called SCs or respirasomes (Schagger and Pfeiffer, 2000; Lapuente-Brun et al., 2013; Acin-Perez and Enriquez, 2014; Letts et al., 2016). The majority of respiratory complex I is found bound with a complex III dimer and complex IV (I + CIII_2_+CIV), forming a megacomplex structure that contains all complexes required to pass electrons from NADH to O_2_ (Schagger and Pfeiffer, 2000). Alternatively, a Supercomplex, containing all the complexes of the electron transport chain (I_2_II_2_III_2_IV_2_) also exists (Guo et al., 2017a). The SCs differ in their composition and stoichiometry (Acin-Perez et al., 2008; Wittig and Schagger, 2009). BN-PAGE is an effective experimental strategy to characterize the organization of the SC/MC structures (Chaban et al., 2014). BN-PAGE analysis showed that Chrm1 expression was effective in causing a plastic change in the OXPHOS system of the tumor cells. We showed that loss of Chrm1 in the cortical mitochondria had no effect on the relative abundance of OXPHOS proteins but disrupted SCs/MCs and cristae structure, which was associated with reduced mitochondrial function. Growing evidence supports the presence of SC as *in vivo* functional entities localized into the cristae (Cogliati et al., 2016). The efficiency of mitochondria-dependent cell growth depends on the cristae shape (Cogliati et al., 2013). A recent study showed that physical activity increased the stoichiometry of mitochondrial SC content in human skeletal muscle (Greggio et al., 2017). Therefore, Chrm1-mediated loss of SC/MC structures may underlie the observed functional deficits in cortical mitochondria.

One important question is how the deletion of Chrm1 is linked to the observed molecular and physiological phenotypes in cortical mitochondria. The answer to this question requires a detailed analysis of the Chrm1 downstream signaling which is subject to future studies. However, some speculations can be made based on existing evidence. Several studies reported that different components of the GPCR transduction pathway modulate mitochondrial function (Mohammad Nezhady et al., 2020; Fasciani et al., 2022). The presence of several GPCRs, for example, purinergic receptors (Belous et al., 2006), angiotensin II receptors (Abadir et al., 2011; Gwathmey et al., 2012), 5-hydroxytryptamine receptor (Wang et al., 2016), melatonin receptor (Suofu et al., 2017), and cannabinoid receptors (Hebert-Chatelain et al., 2016) have been reported in the mitochondrial membranes modulating mitochondrial function. Therefore, it would not be surprising if future studies report Chrm1 localization on the mitochondrial membrane. Interestingly, a recent study (published in the bioRxiv preprint server) by Fasciani et al. (Fasciani et al., 2021) demonstrated that the carboxy-terminal of Chrm2 protein (a Chrm1 paralog) is translated under the influence of an internal ribosomal entry sequence (IRES) with the truncated protein product containing the transmembrane regions 6 and 7 to be exclusively sorted to the mitochondrial inner membrane where it regulates mitochondrial function. The authors also suggested that the IRES sequence is conserved in other muscarinic receptor subtypes. Therefore, Chrm1 may have mitochondrial localization signals, future studies will unravel this yet unexplored area of muscarinic signaling. Furthermore, it is important to note that different mediators of GPCR signal transduction, for example, trimeric G proteins (Gα_12_, Gα_i_, and Gβ_γ_) (Lyssand and Bajjalieh, 2007; Andreeva et al., 2008; Zhang et al., 2010) and the kinase of the G protein-coupled receptor type 2 (GRK2) (Fusco et al., 2012; Chen et al., 2013) have been reported to be localized in mitochondria. Functional involvement of these GPCR signaling mediators regulating mitochondrial physiology is supported by the observation that activation of β2-adrenergic receptors (GPCRs) by catecholamines translocate GRK2 to the mitochondria, reduces ATP loss, and induces mitochondrial biogenesis (Ciccarelli et al., 2013). Interestingly, GRK2 is a CHRM1 desensitizing enzyme (Haga et al., 1996; Yeatman et al., 2014), therefore, it is possible that CHRM1 signaling may also exert similar effects on mitochondrial physiology through GRK2 translocation. On the other hand, GPCR-β-Arrestin signaling activates extracellular signal-regulated kinases (ERK1/2) (Ahn et al., 2004; Goldsmith and Dhanasekaran, 2007). ERK1/2 has been reported to be localized in mitochondria (Alonso et al., 2004), directly or indirectly regulating a myriad of mitochondrial functions (Javadov et al., 2014; Cook et al., 2017). Thus, there is compelling evidence of a potential link between CHRM1 signaling and the regulation of mitochondrial function. However, the mechanistic details of the cause-and-effect relationship have yet to be delineated.

Loss of Chrm1 dramatically increased (wild-type versus Chrm1^-/-^: 2% versus 82%) the abundance of dark neurons in the cerebral cortex. The observation of light and dark neurons is based on tinctorial properties. Both toluidine staining and TEM preparation identified the darkly stained neurons. Toluidine blue is a basic thiazine metachromatic dye with a high affinity for acidic tissue components, thereby it stains tissues rich in DNA and RNA (Sridharan and Shankar, 2012). On the other hand, TEM preparation involves the use of uranyl acetate, which enhances the contrast (differences in the electron density of the organic molecules) by interaction with lipids and proteins, and lead citrate, which enhances the contrast by interacting with proteins and glycogens (Reynolds, 1963). There is no doubt that the appearance of dark neurons or dark-stained synaptic processes is due to a change in the organic content of the neurons. The important question is what caused such changes in the physiochemical properties of the neurons and what are the functional implications. Alteration in the relative abundance of dark neurons in the mammalian cerebral cortex under different experimental conditions and pathological states has been previously reported (Murakami et al., 1997; Zimatkin and Bon’, 2018; Kherani and Auer, 2008; Akhmadeev and Kalimullina, 2005). For example, inhibition of the Na^+^/K^+^ ATPase pump with Ouabain, a plant-derived cardiac glycoside, caused the appearance of dark neurons in the rat cerebral cortex (Cornog et al., 1967). On the other hand, epilepsy (Soderfeldt et al., 1983), hypoglycemia, and ischemia (Kalimo et al., 1981; Czurko and Nishino, 1993; Kovesdi et al., 2007) are known to give rise to dark neurons in the cortex. There are conflicting viewpoints regarding the cause of the appearance of dark neurons. Some hypothesized dark neurons are apoptotic (Bagheri-Abassi et al., 2015). Dark neurons are not apoptotic cells because neither dark nor light neurons from the human somatosensory cortex reacted to nick end labeling for the detection of DNA damage (Murakami et al., 1997). Besides, it has been demonstrated that around 90%-99% of dark neurons recover (reversible type) after some time, while in contrast, only a few proportions of dark neurons become dead neurons (Csordás et al., 2003). Some may argue that mechanical trauma to the brain before fixation may produce dark neurons (Cammermeyer, 1978), but if that is true then one would expect to see the same frequency of dark neurons in the wild type and Chrm1^-/-^ mouse. Our finding of Chrm1 signaling loss and the appearance of dark neurons points to the role of defective neurotransmission as a causal factor for the appearance of dark neurons. It is important to note that all conditions reported previously causing the appearance of dark neurons either disturb neuronal ion gradients (Ouabain) or trigger the release of excessive neurotransmitter (glutamate in epilepsy) (Sarlo and Holton, 2021), or excitatory aspartate/glutamate (hypoglycemia/ischemia) (Drejer et al., 1985; Burke and Nadler, 1989) indicating a pharmacologic origin of dark neurons. Interestingly, it has been demonstrated that treatment with pharmacological agents targeted to N-methyl-D-aspartate (NMDA) abolished dark neurons in rat cortex (Kherani and Auer, 2008). It has been reported that Chrm1 is highly expressed in the glutamatergic pyramidal neurons in the mouse cerebral cortex (Yamasaki et al., 2010) and ACh-signaling modulates NMDA-mediated neurotransmission in cortical neurons (Aramakis et al., 1997). Therefore, it is tempting to hypothesize that loss of Chrm1 affected glutamatergic responses in the mouse cortex causing the appearance of dark neurons. Experiential validation of this aspect of Chrm1 signaling is subject to future studies.

This study provides novel insight into the hitherto unknown effect of Chrm1 loss that leads to an alteration in multiple physiological and ultrastructural properties of cortical mitochondria that are highly relevant to understanding the molecular basis of cholinergic hypofunction and Alzheimer’s pathogenesis (Clader and Wang, 2005; Jiang et al., 2014). Swollen mitochondria with distortion and severe loss of cristae, with less dense matrix have been observed in the neuropil in triple transgenic AD (3xTg-AD) mice (Martins et al., 2017) as well as human *postmortem* brain tissues from AD patients (Hirai et al., 2001b). The defective mitochondrial cristae structure under Chrm1 loss condition may have been due to altered ATP synthase oligomerization and SC/MC assembly leading to respiratory deficits. The exact mechanism connecting Chrm1-signaling with the regulation of mitochondrial structural/functional phenotypes is not yet known, but recent microarray-based gene expression analysis revealed a wide range of genes encoding mitochondrial proteins are altered in Chrm1^-/-^ mouse cortex compared to wild-type (Dean and Scarr, 2021). Our identification of Chrm1 signaling loss as a causal factor leading to mitochondrial structural and functional abnormalities will set the direction for future research. Delineation of the detailed molecular pathway downstream of Chrm1 signal transduction is essential for therapeutic intervention of mitochondrial abnormalities in AD.

## Data Availability

The original contributions presented in the study are included in the article/Supplementary Material, further inquiries can be directed to the corresponding author.

## Abbreviations

OXPHOS, Oxidative phosphorylation; mAChR, Muscarinic acetylcholine receptors; Chrm1, Cholinergic Receptor Muscarinic 1; C, Complex; GPCR, G protein-coupled receptor; AD, Alzheimer’s disease; ACh, Acetylcholine; TEM, Transmission electron microscope; OCR, Oxygen consumption rate.
