# Supplementary figures and images for: Loss of cholinergic receptor muscarinic 1 impairs cortical mitochondrial structure and function: implications in Alzheimer’s disease
Source: Front Cell Dev Biol. 2023 May 18;11:1158604. doi: 10.3389/fcell.2023.1158604 (PMC10233041; doi:10.3389/fcell.2023.1158604)

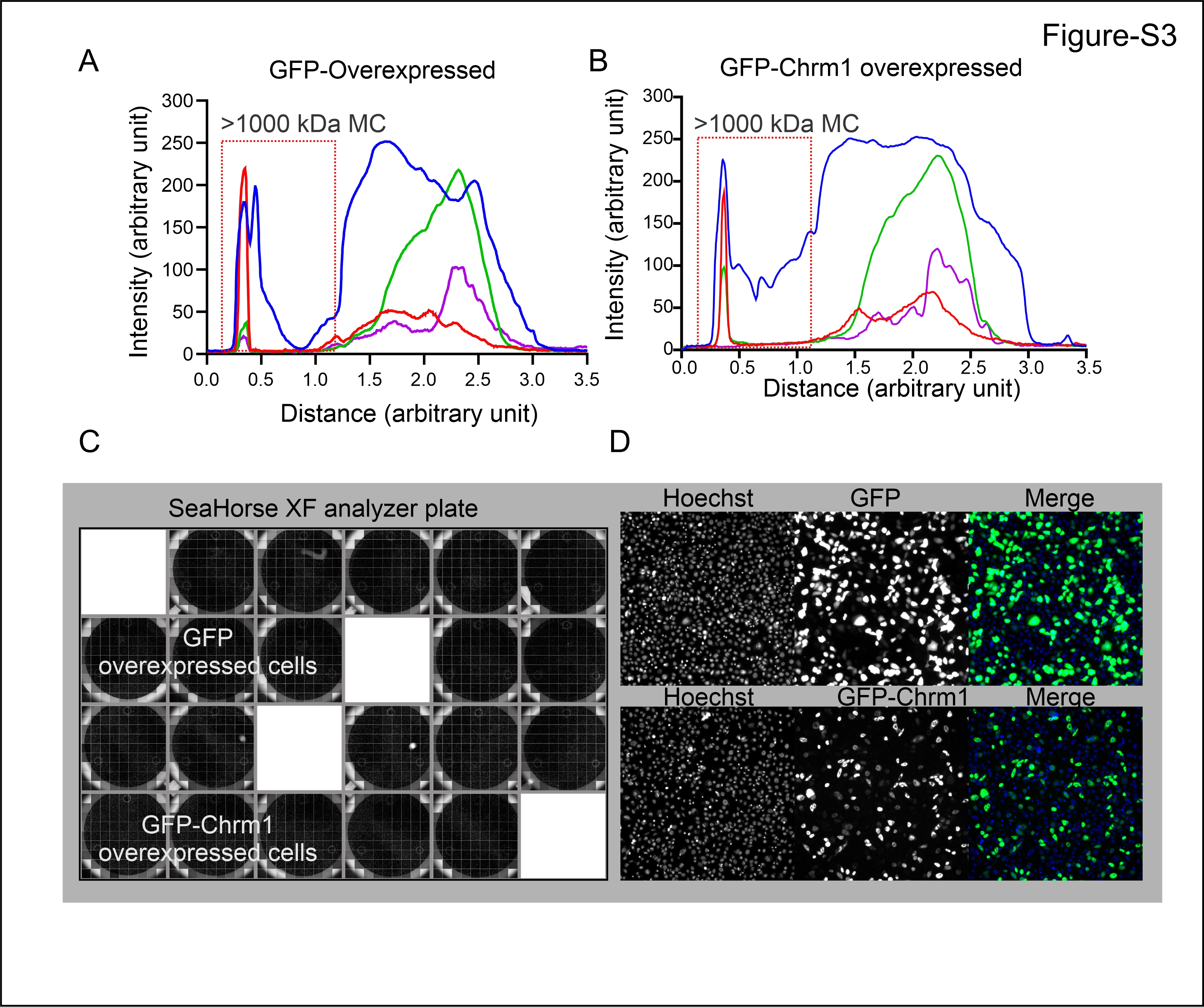

Supplement: Supplementary file 1 [file Image3.JPEG]

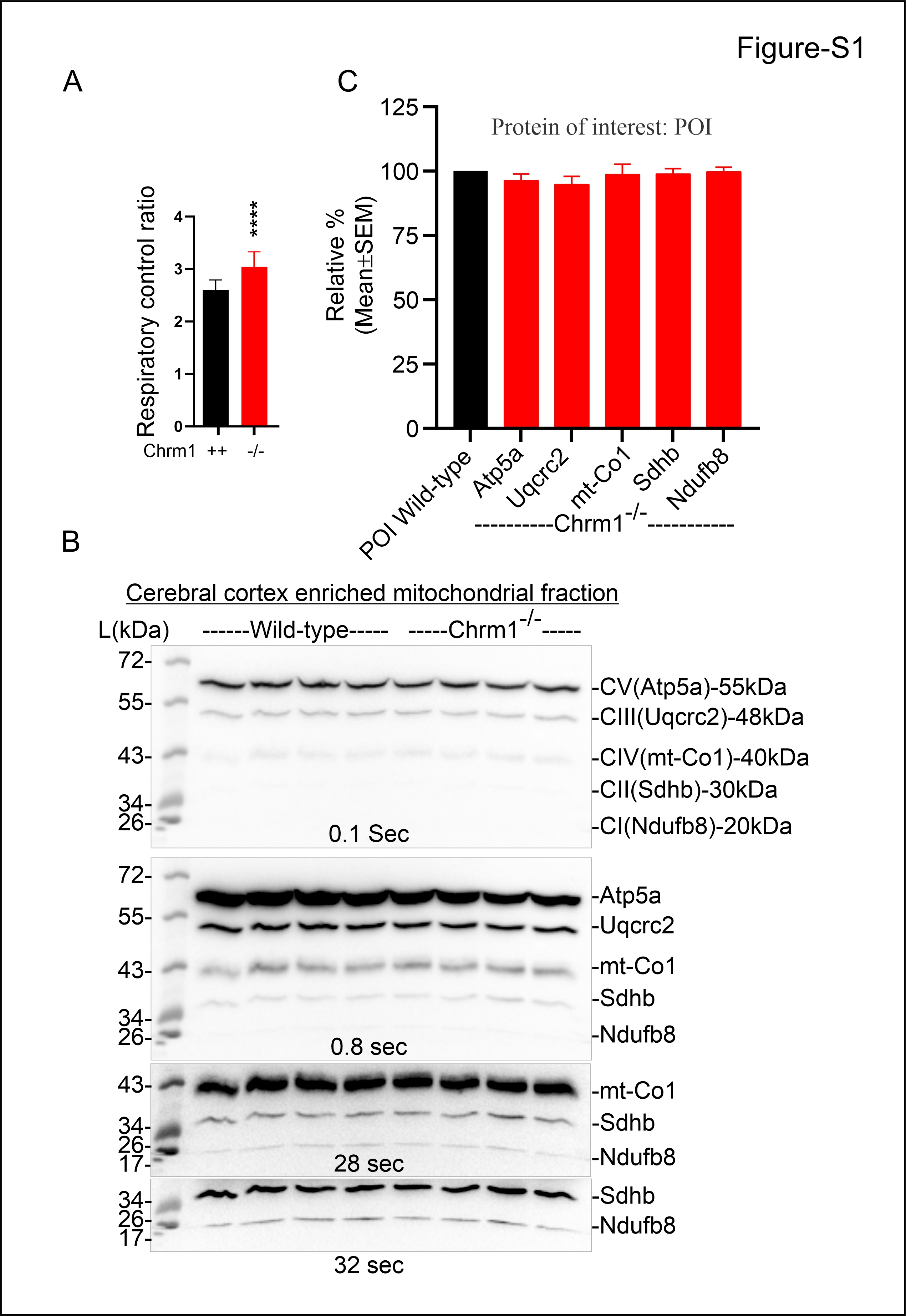

Supplement: Supplementary file 3 [file Image1.JPEG]

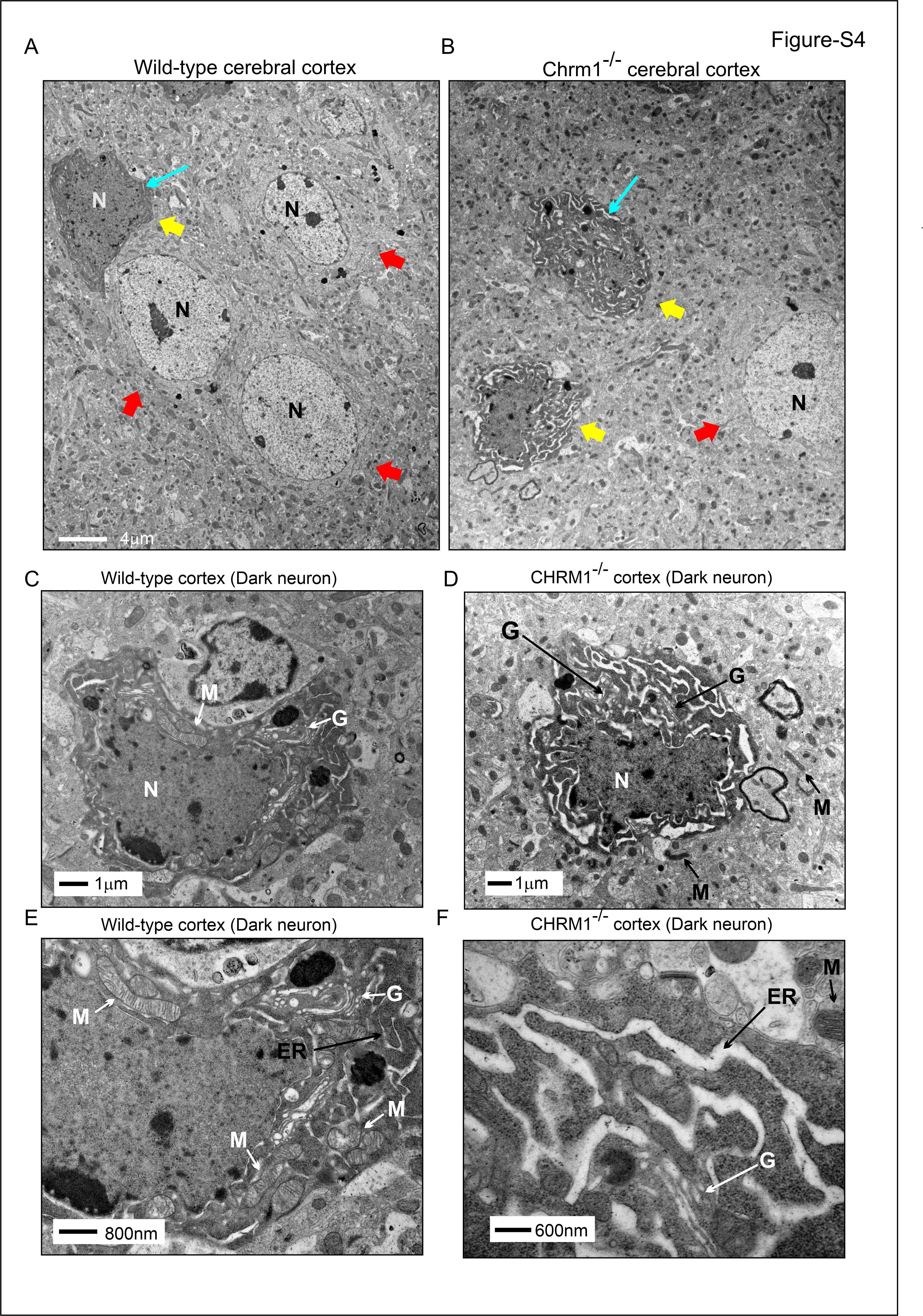

Supplement: Supplementary file 4 [file Image4.JPEG]

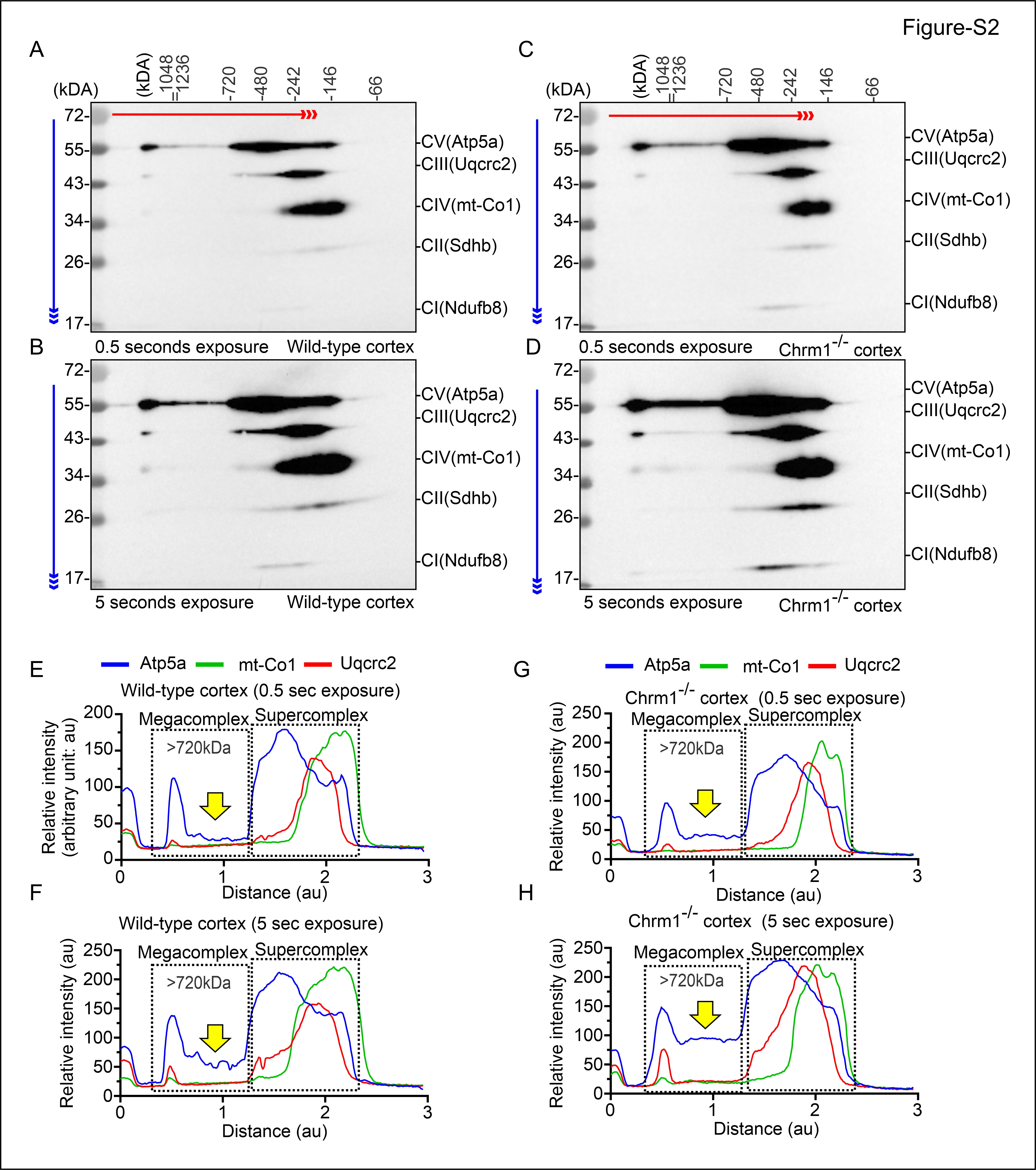

Supplement: Supplementary file 5 [file Image2.JPEG]

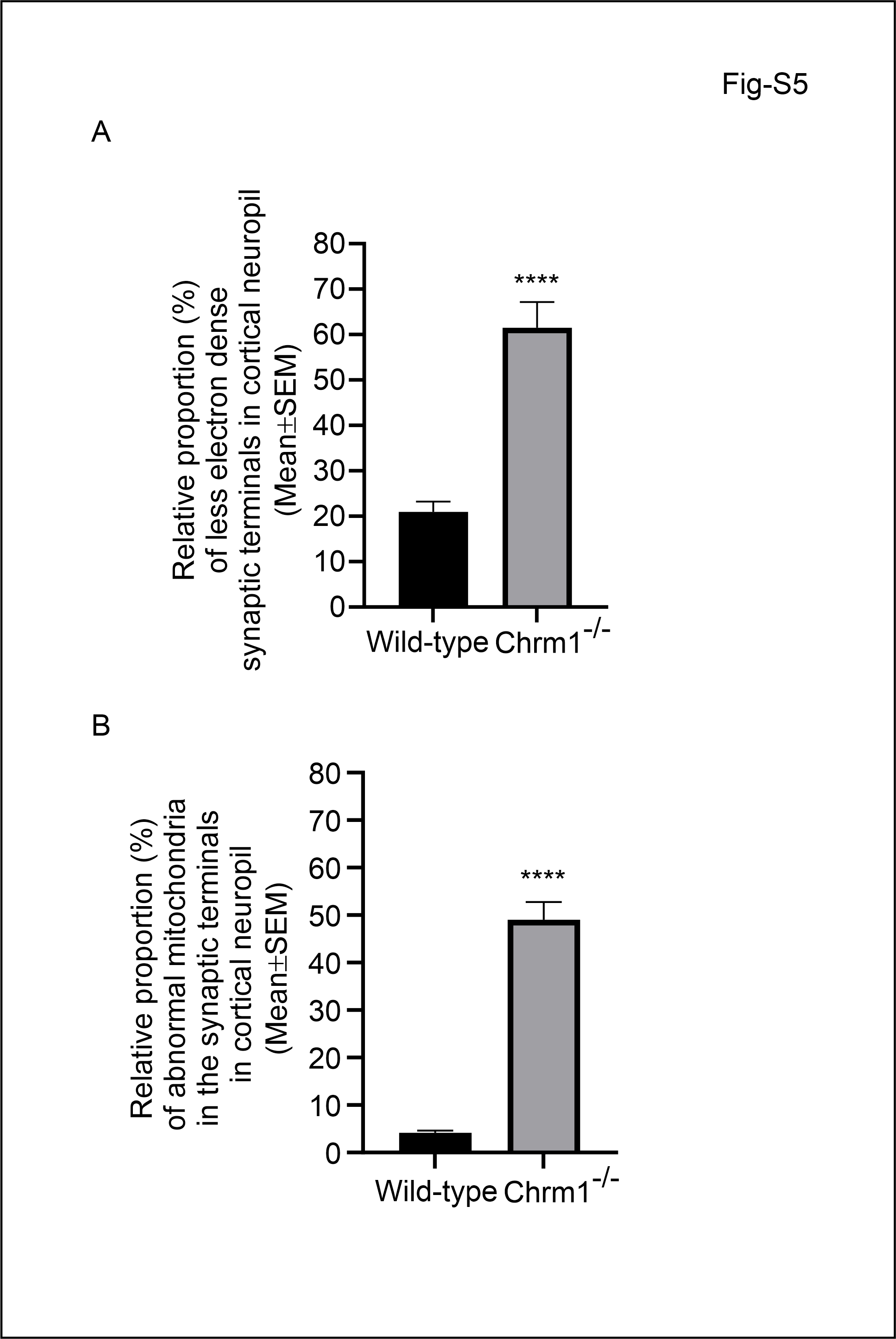

Supplement: Supplementary file 6 [file Image5.JPEG]
