# Supplementary material for: Loss of cholinergic receptor muscarinic 1 impairs cortical mitochondrial structure and function: implications in Alzheimer’s disease
Source: Front Cell Dev Biol. 2023 May 18;11:1158604. doi: 10.3389/fcell.2023.1158604 (PMC10233041; doi:10.3389/fcell.2023.1158604)
Supplement: Supplementary file 2 [file Table1.DOCX]

**Table 1**: List of antibodies.

| Antibody | Catalog number | Vendor | Type | Lot number |
| --- | --- | --- | --- | --- |
| Chrm1 | Sc-365966(G-9) | Santa Cruz Biotechnology | Mouse monoclonal | F2812 |
| GFP | Sc-9996 | Santa Cruz Biotechnology | Mouse monoclonal | G1615 |
| OXPHOS-rodent | MS604-300 | Abcam | Mouse monoclonal | K2342 |
| Vdac1 | Sc-390996 | Santa Cruz Biotechnology | Mouse monoclonal | D1918 |
